# Secret Communication Systems Using Chaotic Wave Equations with Neural Network Boundary Conditions

**DOI:** 10.3390/e23070904

**Published:** 2021-07-16

**Authors:** Yuhan Chen, Hideki Sano, Masashi Wakaiki, Takaharu Yaguchi

**Affiliations:** Graduate School of System Informatics, Kobe University, Kobe 657-8501, Japan; sano@crystal.kobe-u.ac.jp (H.S.); wakaiki@ruby.kobe-u.ac.jp (M.W.); yaguchi@pearl.kobe-u.ac.jp (T.Y.)

**Keywords:** chaotic synchronization, secret communication system, van der Pol boundary condition, deep learning

## Abstract

In a secret communication system using chaotic synchronization, the communication information is embedded in a signal that behaves as chaos and is sent to the receiver to retrieve the information. In a previous study, a chaotic synchronous system was developed by integrating the wave equation with the van der Pol boundary condition, of which the number of the parameters are only three, which is not enough for security. In this study, we replace the nonlinear boundary condition with an artificial neural network, thereby making the transmitted information difficult to leak. The neural network is divided into two parts; the first half is used as the left boundary condition of the wave equation and the second half is used as that on the right boundary, thus replacing the original nonlinear boundary condition. We also show the results for both monochrome and color images and evaluate the security performance. In particular, it is shown that the encrypted images are almost identical regardless of the input images. The learning performance of the neural network is also investigated. The calculated Lyapunov exponent shows that the learned neural network causes some chaotic vibration effect. The information in the original image is completely invisible when viewed through the image obtained after being concealed by the proposed system. Some security tests are also performed. The proposed method is designed in such a way that the transmitted images are encrypted into almost identical images of waves, thereby preventing the retrieval of information from the original image. The numerical results show that the encrypted images are certainly almost identical, which supports the security of the proposed method. Some security tests are also performed. The proposed method is designed in such a way that the transmitted images are encrypted into almost identical images of waves, thereby preventing the retrieval of information from the original image. The numerical results show that the encrypted images are certainly almost identical, which supports the security of the proposed method.

## 1. Introduction

Innovations in information processing technology have led to the rapid development of technologies for accessing and transmitting information in recent years, which has greatly improved the convenience of our daily lives. On the other hand, a wide variety of information is exchanged in public through the Internet and other media, so encryption technology has become increasingly important to protect this information.

In this paper, we consider confidential communication methods using chaos synchronization as one of the encryption techniques. Applying the diversity of chaotic series, pseudo-random series can be generated with statistical properties close to those of ideal random series, which can hide the original information. The problem of designing confidential communication systems using chaotic synchronization has been studied since the 1990s. The core idea of the method is to embed communication information into a signal that behaves chaotically. The method accomplishes the steganography and the recovery of communication information [1,2,3,4,5]. For example, by applying the Hénon mapping, a chaotic synchronous control method for discrete-time nonlinear systems can be designed with significant results [4]. A Hénon map (Hénon–Pomeau attractor/map) is a discrete-time dynamical system that can generate chaotic phenomena, with an iterative expression of:

(1) $$\begin{array}{c} \left\{\begin{array}{c} x_{n+1}=1-ax_{n}^{2}+y_{n}\\ y_{n+1}=bx_{n}, \end{array}\right. \end{array}$$

where a,b are parameter values. When they are set to appropriate values, the system will exhibit chaotic behaviors. In [6], the color image encryption algorithms applying scrambling-diffusion are improved by introducing the transforming-scrambling-diffusing model so that the methods have better security and cryptography characteristics. In [7], a SystemC implementation of a chaos-based crypto-processor for the AES algorithm is presented, where the properties of chaotic systems are employed to cope with the parameters of the AES algorithm. An image encryption algorithm is proposed in [8], where the Arnold chaos sequence and the modified AES algorithm are combined. In [9], a method to encrypt multiple images is proposed by a combination of a fast chaotic encryption algorithm and the AES algorithm. A family of complex variable chaotic systems are used to develop an image encryption algorithm in [10]. In [11], hyper-chaos systems and DNA sequences are combined for encrypting images, where the pseudo-random sequence generated by a hyper-chaos system is transformed into a DNA sequence to diffuse the image blocks.

However, in most of the above methods, the information on the chaotic systems must be shared for encryption and decryption to generate the chaotic sequences. In other words, the parameters of the system and the initial conditions for defining the state of the system essentially played the role of the keys. Although the algorithms are quite secure under the assumption that the information on the chaotic system can be shared safely, there remains a risk of information leakage if this is not the case. Several papers have proposed methods to address this problem by using chaotic synchronization [3,12,13,14,15,16,17]. The chaotic systems have positive Lyapunov exponents, which means that small differences in the initial conditions will grow exponentially. Therefore, the phenomenon of synchronization of such systems is surprising; however, it is known that such synchronization can actually occur [18] and much attention has been focused on this phenomenon. If the chaotic systems of the receiver and the sender can be synchronized by introducing appropriate control signals, the sharing of the parameters and the initial conditions becomes unnecessary. In particular, in [3], chaos-based synchronized dynamic keys are designed and an improved chaos-based advanced encryption standard (AES) algorithm is developed so that a powerful method is proposed that solves the problem of using a static key for AES, while retaining the advantages of the chaos synchronization-based method.

We employ a different approach to these methods, in which, while the problem of having to share the initial conditions is addressed by the chaos synchronization technique, that of having to share the parameters of the systems is overcome by introducing extremely complex parameterization of the chaotic systems, that is, neural networks. More precisely, we use certain chaotic phenomena in distributed systems with nonlinear boundary conditions represented by a certain neural network. The neural network is pre-trained so that it defines a chaotic function. As far as the networks are chaotic, neural networks with arbitrary architectures can be used. Therefore, the number of the neural networks, and hence the size of the key space, can be arbitrarily large by enlarging the network architecture.

The construction of a confidential communication system using chaotic phenomena in distributed systems has also been studied before but without the neural-network boundary condition. In this paper, we focus on the system proposed in [19], in which a certain initial boundary value problem of the linear wave equation is employed on a one-dimensional bounded interval, with a linear homogeneous boundary condition at the left end and the nonlinear boundary condition, which has a cubic nonlinearity of the van der Pol type [20,21]. The interaction of these linear and nonlinear boundary conditions leads to chaos in the Riemann invariants (u,v) of the wave equation when the parameters satisfy certain requirements. By constructing an observer by applying the method of characteristics, the appropriate range of the feedback gain is obtained so that the convergence of the dynamics that describes the synchronization error between two mappings is ensured. Through the numerical computation, it is also confirmed that the one-dimensional wave equation with the van der Pol type boundary conditions exhibits a spatio-temporal chaotic behavior in its dynamics [22]. In [19], this chaotic vibration of the wave equation under the van der Pol boundary condition is specifically used to construct the synchronous system. There are two features of this approach—firstly, the synchronization system is easy to construct and secondly, it transmits vector-valued signals in a secure communication system [19].

On the other hand, many information security techniques are based on artificial intelligence. As the problem of image recognition is a specialty of neural networks, neural networks are often used in information security technologies to solve problems of image recognition and image analysis. Biometric technologies, such as face recognition systems and fingerprint authentication, are examples of this [23]. Meanwhile, in terms of security performance, deep learning also does not have the complete capability to guarantee absolute security and privacy. There are many types of attacks that are made on deep learning, such as Causative Attacks, Exploratory Attacks and Indiscriminate Attacks [24]. With these attacks, the model information or the knowledge of the training data can be extracted; these are known as model inversion, model extraction and membership inference, where the attackers steal the training data and produce the expected results, or provide incorrect training data. This means that the attackers may have the ability to change the inputs to the training data, which becomes the reason for parameter changes in the learning model, leading to a significant decrease in the performance of the subsequent classification tasks. To address these leakage risks, privacy-preserving learning, such as Defensive Distillation, has been developed for defending against poisoning attacks; however, these approaches cannot eliminate all security risks [24] at this time.

In this paper, we combine chaos theory and deep learning to construct a model that makes it difficult to steal information and that has a stronger secrecy effect, and apply it to a confidential communication system for color images.

This research aims to provide an information communication system with high secrecy performance, which uses the wave equation with chaotic behaviors and also deep neural networks. More precisely, the proposed approach is to apply a synchronous pair of chaotic distribution systems to encrypt the transmitted object, while applying a deep neural network as a black box to encrypt and decrypt color image information. The following is a brief description of the proposed approach.

**Figure 1:**
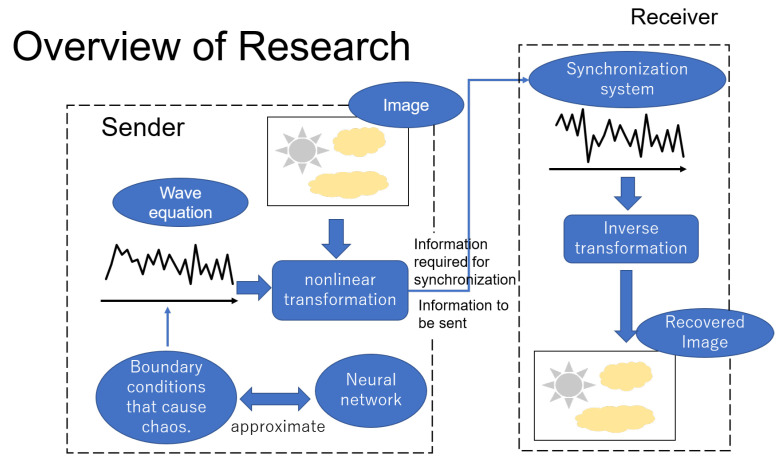
A diagrammatic representation of the research ideas and methods used in this study. It succinctly shows the overall structure of the confidential communication system as well as the design principles, in which the input portrait was created manually by the author and is only used here as an example to assist in illustration.

Firstly, the proposed approach employs the chaotic wave equation, which is an evolutional partial differential equation and hence has two axes (space and time). This feature of the equation enables the method to transmit images. Secondly, the proposed secret communication system consists of two parts, the sender and the receiver, as shown in Figure 1. First, the wave equation on the sender side is under the van der Pol boundary conditions that can cause chaotic phenomena, and a deep neural network is trained so that it has the same chaotic effect. Then, the color images are encrypted by applying a certain nonlinear transformation together with the solutions to the wave equations. The nonlinear transformation is designed so that the inverse of it is computable if the solutions to the wave equations, which are essentially the secret keys, are known. Second, a chaotic synchronization system is established at the receiver side to obtain the decrypted recovered images by inverting the function.

**Figure 2:**
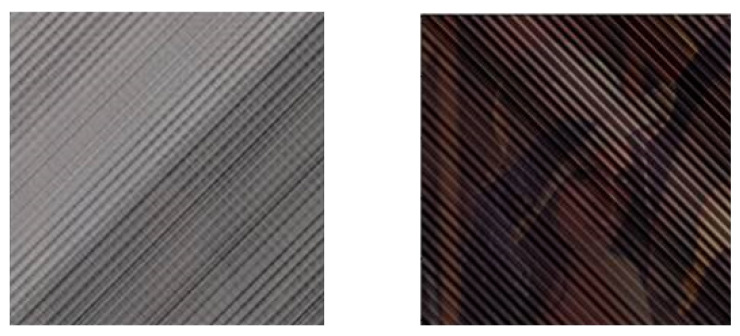
The modulated secrecy image and the restored image after the leakage of some of the parameters of the confidential communication system. The figure on the left is the confidential information after it has been encrypted, and we can see that we have no way of knowing the information about the original image, so we can say that it was a successful secret communication. The figure on the right is the restored image after the leakage of some of the parameters of the confidential communication system; we can almost see that it is a picture of a woman wearing a hat, that is to say, the hacker can restore the transmitted information to some extent after breaking some of the parameters.

The innovation of this study is to use a deep neural network that approximates the boundary conditions yielding the chaotic vibrations, which addresses the problem that the previous system is easier to be cracked and makes it easier for the information to be stolen due to too few parameters of the van der Pol boundary condition when the original chaotic synchronization system is used alone. In fact, in the wave equation with the van der Pol boundary conditions, the three parameters required are a,β and η (see Section 2 for the specific expressions). Suppose that a hacker now steals the specific value of β, and the other two (*a* and η) still exist in a hidden state. Suppose also that that person makes an effort to guess the values of *a* and η and tries to break the encrypted information. In Figure 2, we can see that the possibility exists that the whole information of the portrait can be seen to some extent. Although it was mentioned in the above that deep learning techniques are also under threat from security attacks, a stealer will face greater implementation difficulties than a brute force attack when parameters are partially compromised. More notably, the way the proposed approach corresponds the neural network structure with the boundary conditions at the left end and the right end will also increase the difficulty for the stealer to crack.

This paper is organized as follows. First, in Section 2, the proposed method for grayscale images is described in detail along with some numerical examples. In Section 3, the method is applied to color images. In Section 4, the encryption effects of the proposed method and the AES are tested, respectively, and the results are analyzed. Concluding remarks are shown in Section 5.

## 2. Grayscale Images as Transmission Objects

Before talking about the system for color images, we will explain the distributed system with chaotic vibrations and synchronization systems using grayscale images as transmitted objects, and numerically test it to obtain the encoded images and the restored images, and compare them with the original images, thereby investigating the feasibility of the proposed approach. Since grayscale images do not involve RGB, the system becomes simpler and hence it is somewhat easier to understand than with color images.

### 2.1. Wave Equation with the van der Pol Boundary Conditions

The system we consider here is a linear PDE but with a nonlinear boundary condition, which is from the van der Pol equation without forcing,

(2) $$\begin{array}{l} \left\{\begin{array}{l} \ddot{x}+\left(-\alpha \dot{x}+\beta {\dot{x}}^{3}\right)+\omega_{0}^{2}x=0,\;\alpha ,\beta ,\omega^{2}>0\\ x\left(0\right)=x_{0},\;\dot{x}\left(0\right)=x_{1},\;x_{0},x_{1}\in R, \end{array}\right. \end{array}$$

where $\omega_{0}$ is the fixed frequency of the corresponding linear harmonic oscillator. The energy of this system is given by

(3) $$\begin{array}{c} E\left(t\right)=\frac{1}{2}\left[\dot{x}{\left(t\right)}^{2}+\omega_{0}^{2}x{\left(t\right)}^{2}\right], \end{array}$$

and the time rate of change of energy is

(4) $$\begin{array}{cc} \frac{d}{dt}E\left(t\right) & =\dot{x}\left(t\right)\left[\ddot{x}\left(t\right)+\omega_{0}^{2}x\left(t\right)\right]\\ \; & =\alpha \dot{x}{\left(t\right)}^{2}-\beta {\dot{x}}^{4}\left(t\right), \end{array}$$

so we get

(5) $$\begin{array}{c} \left\{\begin{array}{c} \geq 0,\;\mathrm{if}\;|\dot{x}|\leq {\left(\frac{\alpha }{\beta }\right)}^{\frac{1}{2}}\\ \leq 0,\;\mathrm{if}\;|\dot{x}|>{\left(\frac{\alpha }{\beta }\right)}^{\frac{1}{2}}, \end{array}\right. \end{array}$$

which shows the self-regulation effect, as the energy will increase when $|\dot{x}|$ is small, and the energy will decrease when $|\dot{x}|$ is large. Therefore, unless the initial condition satisfies $x_{0}=x_{1}=0$ in (2), causing E(t)=0 for all t>0, we can know that E(t) will rise and fall between a certain interval of the $\left(B_{1},B_{2}\right)$. The bounds $B_{1}$ and $B_{2}$ can be determined by the parameters α and β.

Let us describe a wave equation below as

(6) $$\begin{array}{c} \frac{1}{c^{2}}y_{tt}\left(x,t\right)-y_{xx}\left(x,t\right)=0,\;0<x<1,\;t>0, \end{array}$$

which defines the linear PDE that describes a vibrating string on the unit interval (0,1), where c>0 denotes the speed of wave propagation. Because the speed of wave *c* is not an essential parameter, we set c=1. Thus, in this paper, we consider

(7) $$\begin{array}{c} y_{tt}\left(x,t\right)-y_{xx}\left(x,t\right)=0,\;0<x<1,\;t>0. \end{array}$$

At the left-end x=0, we choose the following boundary condition:

(8) $$\begin{array}{c} y_{t}\left(0,t\right)=-\eta y_{x}\left(0,t\right),\;t>0,\;\eta >0,\;\eta \neq 1. \end{array}$$

At the right-end x=1, we assume a nonlinear boundary condition,

(9) $$\begin{array}{c} y_{x}\left(1,t\right)=\alpha y_{t}\left(1,t\right)-\beta y_{t}^{3}\left(1,t\right),\;t>0,\;\alpha ,\beta >0. \end{array}$$

The initial conditions are:

(10) $$\begin{array}{c} y\left(x,0\right)=y_{0}\left(x\right),\;y_{t}\left(x,0\right)=y_{1}\left(x\right),\;0\leq x\leq 1 \end{array}$$

for two given smooth functions $y_{0}$ and $y_{1}$.

As a summary, the wave equation with the van der Pol boundary condition is given by

(11) $$\begin{array}{c} \left\{\begin{array}{c} y_{tt}\left(x,t\right)-y_{xx}\left(x,t\right)=0,\;0<x<1,\;t>0\\ y_{x}\left(1,t\right)=\alpha y_{t}\left(1,t\right)-\beta y_{t}^{3}\left(1,t\right),\;t>0\\ y_{t}\left(0,t\right)=-\eta y_{x}\left(0,t\right),\;t>0\\ y\left(x,0\right)=y_{0}\left(x\right),\;y_{t}\left(x,0\right)=y_{1}\left(x\right),\;0\leq x\leq 1, \end{array}\right. \end{array}$$

where we set α,β,η>0 and η≠1. We use the method of characteristics to rewrite (11). Let *u* and *v* be the Riemann invariants [25] of (11)

(12) $$\begin{array}{c} \left\{\begin{array}{c} u\left(x,t\right)=\frac{1}{2}\left\{y_{x}\left(x,t\right)+y_{t}\left(x,t\right)\right\}\\ v\left(x,t\right)=\frac{1}{2}\left\{y_{x}\left(x,t\right)-y_{t}\left(x,t\right)\right\}, \end{array}\right. \end{array}$$

with initial conditions

(13) $$\begin{array}{c} \left\{\begin{array}{c} u\left(x,0\right)=u_{0}\left(x\right)=\frac{1}{2}\left[y_{0}^{\prime }\left(x\right)+y_{1}\left(x\right)\right]\\ v\left(x,0\right)=v_{0}\left(x\right)=\frac{1}{2}\left[y_{0}^{\prime }\left(x\right)-y_{1}\left(x\right)\right] \end{array}\right.\;0\leq x\leq 1. \end{array}$$

Then, the substitution of *u* and *v* into the boundary condition (9) gives

(14) $$\begin{array}{c} u\left(1,t\right)=F_{\alpha ,\beta }\left(v\left(1,t\right)\right),\;t>0. \end{array}$$

Besides, since $y_{x}\left(1,t\right)=u\left(1,t\right)+v\left(1,t\right)$, $y_{t}\left(1,t\right)=u\left(1,t\right)-v\left(1,t\right)$ at the right end x=1, the relationship (14) becomes

(15) $$\begin{array}{c} \beta {\left(u-v\right)}^{3}+\left(1-\alpha \right)\left(u-v\right)+2v=0. \end{array}$$

Thus, let us summarize what the 1st order hyperbolic equation of (13) looks like after using Riemann invariants *u* and *v*.

(16) $$\begin{array}{c} \Sigma_{0}:\left\{\begin{array}{c} u_{t}\left(x,t\right)=u_{x}\left(x,t\right),\;x\in \left(0,1\right),\;t>0\\ v_{t}\left(x,t\right)=-v_{x}\left(x,t\right),\;x\in \left(0,1\right),\;t>0\\ u\left(1,t\right)=F_{\alpha ,\beta }\left(v\left(1,t\right)\right),\;t>0\\ v(0,t)=qu(0,t),\;t>0\\ u\left(x,0\right)=\frac{1}{2}\left[y_{0}^{\prime }\left(x\right)+y_{1}\left(x\right)\right]=u_{0}\left(x\right),\;x\in \left[0,1\right]\\ v\left(x,0\right)=\frac{1}{2}\left[y_{0}^{\prime }\left(x\right)-y_{1}\left(x\right)\right]=v_{0}\left(x\right),\;x\in \left[0,1\right], \end{array}\right. \end{array}$$

where $q=\frac{1+\eta }{1-\eta }$ by (12), from which follows

(17) $$\begin{array}{c} v\left(0,t\right)=q\left(u\left(0,t\right)\right)=\frac{1+\eta }{1-\eta }u\left(0,t\right),\;t>0. \end{array}$$

**Figure 3:**
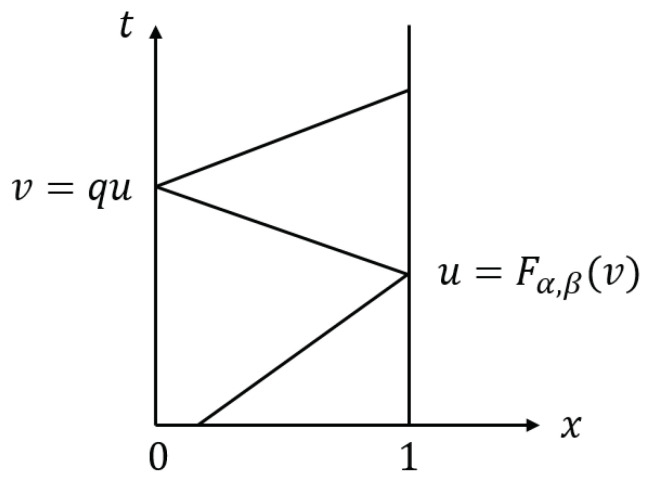
Reflection of the characteristic lines. The waves are reflected at x=0 and x=1, where the two functions v=qu and $u=F_{\alpha ,\beta }\left(v\right)$ are applied.

(15) and (17) represent the boundary conditions of the two boundaries at which the waves are reflected, respectively, as shown in Figure 3. When the wave reaches the right end x=1, it is reflected to the left via (15) changing direction; when it reaches the left end, it is transmitted to the right via (17).

For (15), with 0<α≤1, there exists a unique u∈R corresponding to each v∈R [20]. On the other hand, when α>1, for each v∈R, in general there exist two or three distinct u∈R, satisfying (15). Thus, in the latter case, $u=F_{\alpha ,\beta }\left(v\right)$ is not well-defined, and hence the original PDE system (11) is not unique.

For parameters, for example, α=0.5,β=1 and η=0.625 are used, but special attention should be paid to the setting of η, which has theoretically been shown to exponentially increase the total variation of the system if it is chosen as an appropriate value. Since total variation is one of the indicators of the severity of the function change, its increase means that the system behaves chaotically.

So far, we have obtained a system of PDEs, which is a wave equation that can cause chaotic vibrations. This system allows us to chaoticize the transmitted image, and in order to restore the chaotic image in a later step, we need to construct a synchronization system. This system is designed so that the system exhibits exactly the same chaotic vibrations of the original system. In the next section, we will construct such a synchronization system.

### 2.2. Synchronization System

**Figure 4:**
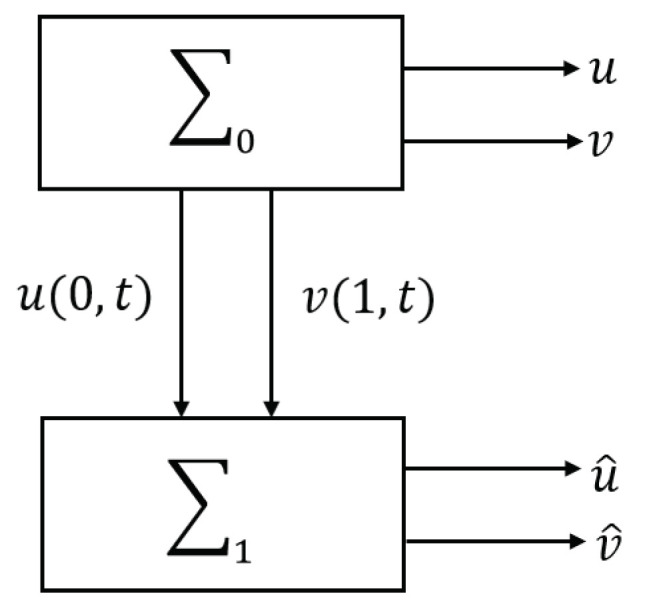
Synchronization system $\sum_{1}$.

The construction of a synchronization system requires a portion of the original system’s information. Hence, the sender sends two signals to the receiver: one is a secret, coded image, and the other is the signal needed for the synchronized system to restore the original system’s state. For the system $\sum_{0}$ (16), consider the following system $\sum_{1}$:

(18) $$\begin{array}{c} \Sigma_{1}:\left\{\begin{array}{c} {\hat{u}}_{t}\left(x,t\right)={\hat{u}}_{x}\left(x,t\right),\;x\in \left(0,1\right),\;t>0\\ {\hat{v}}_{t}\left(x,t\right)=-{\hat{v}}_{x}\left(x,t\right),\;x\in \left(0,1\right),\;t>0\\ \hat{u}\left(1,t\right)=F_{\alpha ,\beta }\left(v\left(1,t\right)\right),\;t>0\\ \hat{v}\left(0,t\right)=qu\left(0,t\right),\;t>0\\ \hat{u}\left(x,0\right)={\hat{u}}_{0}\left(x\right),\;x\in \left[0,1\right]\\ \hat{v}\left(x,0\right)={\hat{v}}_{0}\left(x\right),\;x\in \left[0,1\right], \end{array}\right. \end{array}$$

with two signals, u(0,t),v(1,t), as inputs. The relationship between the two systems is shown in Figure 4. We set variables $\tilde{u}=u-\hat{u}$, $\tilde{v}=v-\hat{v}$ to obtain the error system as follows:

(19) $$\begin{array}{c} \left\{\begin{array}{c} {\tilde{u}}_{t}\left(x,t\right)={\tilde{u}}_{x}\left(x,t\right),\;x\in \left(0,1\right),\;t>0\\ {\tilde{v}}_{t}\left(x,t\right)=-{\tilde{v}}_{x}\left(x,t\right),\;x\in \left(0,1\right),\;t>0\\ \tilde{u}\left(1,t\right)=0,\;t>0\\ \tilde{v}\left(0,t\right)=0,\;t>0\\ \tilde{u}\left(x,0\right)={\tilde{u}}_{0}\left(x\right),\;x\in \left[0,1\right]\\ \tilde{v}\left(x,0\right)={\tilde{v}}_{0}\left(x\right),\;x\in \left[0,1\right]. \end{array}\right. \end{array}$$

Solving the system using the method of characteristics, we can see that, at any initial value ${\tilde{u}}_{0}$, ${\tilde{v}}_{0}$, the solution $\tilde{u}\left(\;\cdot \;,t\right)$ and $\tilde{v}\left(\;\cdot \;,t\right)$ is completely zero at moment t=2. In other words, at moment t=0, there is no error between system (18) and system (16) and they reach a synchronized state.

In the previous study [19], it was assumed that the parameters of the system and information about the boundary conditions necessary to define (18) are shared in advance. The sender then sends the modulated information using the states of the chaotic system for secret communication, while the receiver sends the values u(0,t) and v(1,t) at the boundary required for synchronization at the sender to the synchronization system to evolve and demodulate the information by using the system state used during modulation.

### 2.3. Proposed Secret Communication System

In this study, we use neural networks to improve the security of the method by black-boxing the boundary conditions. Since the experimental objects in this section are grayscale images, the images can be transmitted as a single signal through the secure communication system. In the paper [19], in particular, the modulation and demodulation of the (M+1)×(L+1) pixel image data are studied by extending the method of the paper [4] to the distribution system. For this purpose, system (16) and synchronization system (18) must be discretized in both spatial and temporal directions.

**Figure 5:**
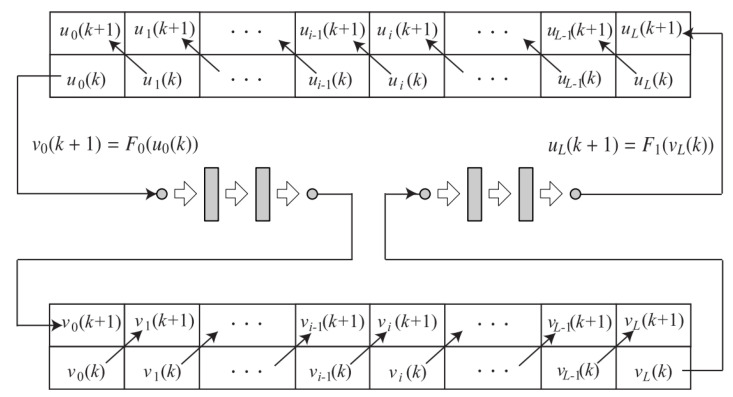
Time evolution of u(k) and v(k) in the discretized systems with the boundary conditions given by the neural networks.

Divide the interval [0,1] equally into *L* parts and set the division points $x_{i}=i\Delta x\left(i=0,1,\cdots ,L\right)$. Here, the interval is given by $\Delta x=\frac{1}{L}$. The time step size is set to Δt and we write $t_{k}=k\Delta t\left(k=0,1,\cdots \right)$. For the system (16) u(x,t),v(x,t) denote the states, $u_{i}\left(k\right),v_{i}\left(k\right)$ denote the approximation values of $u\left(x_{i},t_{k}\right),v\left(x_{i},t_{k}\right)$ on the grid points $\left(x_{i},t_{k}\right)$. For simplicity, we introduce the (L+1) dimension vectors $u\left(k\right)={\left[u_{0}\left(k\right),u_{1}\left(k\right),\cdots ,u_{L}\left(k\right)\right]}^{T}$, $v\left(k\right)={\left[v_{0}\left(k\right),v_{1}\left(k\right),\cdots ,v_{L}\left(k\right)\right]}^{T}$. Denote ${\hat{u}}_{i}\left(k\right)=\hat{u}\left(x_{i},t_{k}\right)$, ${\hat{v}}_{i}=\hat{v}\left(x_{i},t_{k}\right)$ for system (18) as well, and denote the (L+1) dimension vector $\hat{u}\left(k\right)=[{\hat{u}}_{0}\left(k\right)$, ${\hat{u}}_{1}\left(k\right),\cdots ,{\hat{u}}_{L}{\left(k\right)]}^{T}$, $\hat{v}\left(k\right)={\left[{\hat{v}}_{0}\left(k\right),{\hat{v}}_{1}\left(k\right),\cdots ,{\hat{v}}_{L}\left(k\right)\right]}^{T}$. Thus, for example, in Figure 4, the signals u(0,t),v(1,t) sent from $\sum_{0}$ to $\sum_{1}$ for synchronization are discretized to $u_{0}\left(k\right),v_{L}\left(k\right)$, respectively. The time evolution of the vector u(k),v(k) is shown in Figure 5. We approximate the $\sum_{0}$ and $\sum_{1}$ by the upwind difference using the same step sizes in the spatial and temporal directions so that the CFL number is 1. As a result, we obtain an algorithm where $u_{i}\left(k\right)$ is transported to $u_{i-1}\left(k+1\right)$ and $v_{i-1}\left(k\right)$ is to $v_{i}\left(k+1\right)$. More precisely, the system after discretization is

(20) $$\begin{array}{c} \left\{\begin{array}{c} \frac{u_{i}\left(k+1\right)-u_{i}\left(k\right)}{\Delta t}=\frac{u_{i+1}\left(k\right)-u_{i}\left(k\right)}{\Delta x},\;k>0,i\in \left\{1,2,\cdots ,L-1\right\}\\ \frac{v_{i}\left(k+1\right)-v_{i}\left(k\right)}{\Delta t}=-\frac{v_{i}\left(k\right)-v_{i-1}\left(k\right)}{\Delta x},\;k>0,i\in \left\{1,2,\cdots ,L-1\right\}\\ u_{L}\left(k\right)=F_{\alpha ,\beta }\left(v_{L}\left(k\right)\right),\;k>0,\\ v_{0}\left(k\right)=qu_{0}\left(k\right),\;k>0. \end{array}\right. \end{array}$$

Here, we set $\Delta t=\Delta x=\frac{1}{L}$ as the time and space step sizes, which gives

(21) $$\begin{array}{c} u_{i}\left(k+1\right)=u_{i+1}\left(k\right),\;k>0,i\in \left\{1,2,\cdots ,L-1\right\}\\ v_{i}\left(k+1\right)=v_{i-1}\left(k\right),\;k>0,i\in \left\{1,2,\cdots ,L-1\right\}. \end{array}$$

$\sum_{1}$ is discretized in the same manner. Henceforth, the systems $\sum_{i}\left(i=0,1\right)$ after discretization are denoted by ${\bar{\Sigma }}_{i}\left(i=0,1\right)$.

As we mentioned earlier, due to the risk of theft due to the small number of parameters of the wave Equation (16) with the van der Pol boundary condition, in this study we propose a way to enhance security by approximating the van der Pol boundary condition with a neural network instead of the original boundary condition. The first half of this section describes how the u,v evolve over time, and the two systems $\sum_{0},\sum_{1}$ are discretized. As shown in Figure 5, in the previous method, the waves at the boundaries are updated by $v_{0}\left(k+1\right)=qu_{0}\left(k\right)$ and $u_{L}\left(k+1\right)=F_{\alpha ,\beta }\left(v_{L}\left(k\right)\right)$, respectively. In the proposed method, which uses a neural network instead of these boundary conditions, we need two functions, $F_{1}$ and $F_{0}$, in order to simulate the boundary conditions.

**Figure 6:**
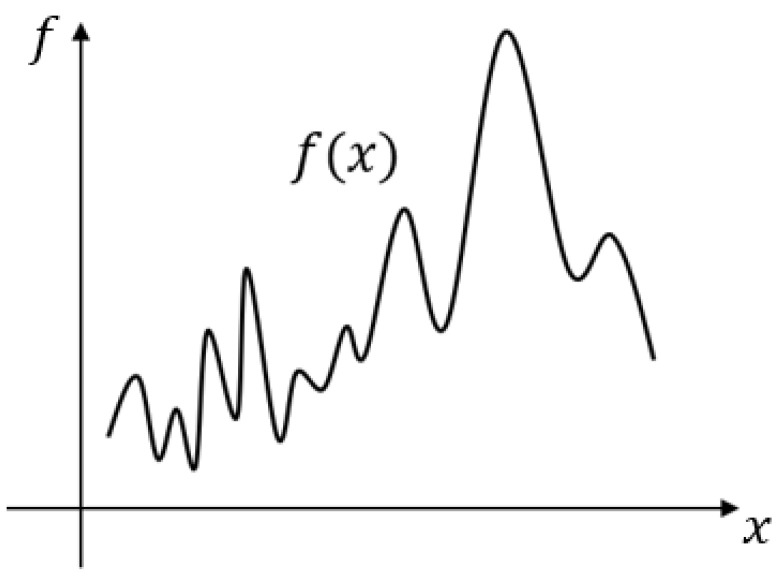
A complex continuous function f(x).

One of the properties about neural networks is that they can approximate any continuous functions defined on compact sets. That is, neural networks can be a complicated, wiggly function, f(x), as in Figure 6.

For every possible input *x*, no matter what function it is, there is a neural network whose output value is close to f(x). This property holds for functions with multiple inputs $f=f(x_{1},...,x_{m})$ and multiple outputs. This property of neural networks is called the universal approximation property. Moreover, this universality theorem holds even for neural networks that have only one hidden layer between the input and output layers. In other words, the expressive power is extremely high even for extremely simple network structures.

**Figure 7:**
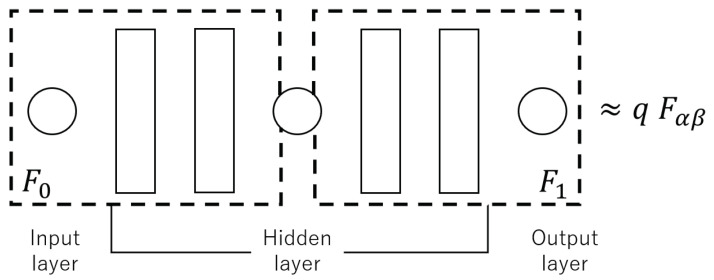
Relationship between the structure of neural networks and boundary conditions.

The neural network applied here is composed of seven layers of perceptrons, as shown in Figure 7, and is approximated to $qF_{\alpha ,\beta }$ by training. Since in this section we suppose that the images are of grayscale, both the input and output layers of the neural network have a single neuron. In this proposed approach, the fourth layer is also designed as a single neuron. The whole neural network is divided into two parts; the first part is the first to fourth layers, which is assumed to represent a function $F_{0}$, and the second part is the fifth to seventh layers, which is assumed to represent a function $F_{1}$. As shown in Figure 5, using the learned neural network, the left boundary condition is replaced, from $v_{0}\left(k+1\right)=qu_{0}\left(k\right)$ to the front half of the neural network $F_{0}$, while the right boundary condition is replaced, from $u_{L}\left(k+1\right)=F_{\alpha ,\beta }\left(v_{L}\left(k\right)\right)$ to the back half of the neural network $F_{1}$. In this way, the boundary conditions are black-boxed and the information becomes difficult to leak.

After approximating the van der Pol boundary condition, we tested the resulting new neural network by computing its Lyapunov exponent. In fact, when dealing with actual chaotic phenomena, instead of providing a clear mathematical definition of chaos, whether the system is chaotic or not is checked by a practical condition, which is determined by a certain criterion that is numerically computable. A dynamical system *F* is chaotic if it satisfies at least one of the following conditions:

(1) *F* has a sensitive dependence on initial conditions within the defined region.
(2) *F* has positive Lyapunov exponents at all points in the definite domain excluding the final immobile point [26,27].

In this study, we use the method of calculating the Lyapunov exponents, that is, condition (2). Lyapunov exponents are used to quantify the separation rate between infinitely close trajectories in a dynamical system. Specifically, under the assumption that linearization is feasible, the separation rate of the two trajectories with an initial interval of $\sigma Z_{0}$ is

(22) $$\begin{array}{c} \left|\sigma Z\left(t\right)\right|\approx e^{\lambda t}\left|\sigma Z_{0}\right|, \end{array}$$

where λ is the Lyapunov exponents. In this section, where the images are assumed to be grayscale, since no color issues are involved, it can be viewed as a one-dimensional discrete-time system, in which case the Lyapunov exponent λ for a one-dimensional map $x_{n+1}=L\left(x_{n}\right)$ is defined by

(23) $$\begin{array}{c} \lambda =\lim_{N\to \infty }\frac{1}{N}\sum_{i=1}^{N-1}\log\left|L^{\prime }\left(x_{i}\right)\right|, \end{array}$$

where x∈R is the state variable of the system and n∈N is the discrete time. If the value of the Lyapunov exponent computed in (23) is positive, then the system *F* is considered to be chaotic. Therefore, we use this method to judge whether the trained neural network successfully approximates the chaotic functions.

**Figure 8:**
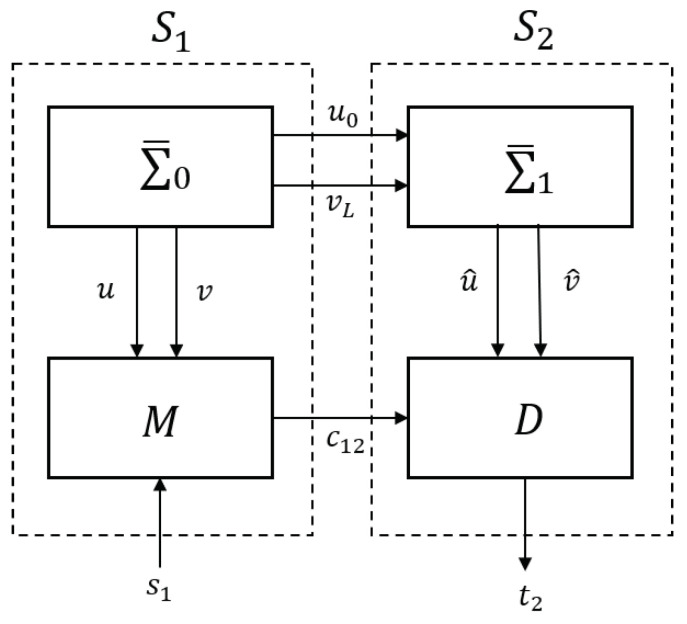
Secure communication system.

As shown in Figure 8, the specific components of the whole secret communication system are the system ${\bar{\Sigma }}_{0}$, synchronization system ${\bar{\Sigma }}_{1}$, modulation *M* and demodulation *D*, where modulation and demodulation, respectively, are represented by the following equations:

**Modulation**

(24) $$\begin{array}{c} M:\left\{\begin{array}{c} w\left(k+1\right)=G(u\left(k\right),v\left(k\right),w\left(k\right),s_{1}\left(k+1\right))\\ c_{12}=w\left(k\right), \end{array}\right. \end{array}$$

**Demodulation**

(25) $$\begin{array}{c} D:t_{2}\left(k+1\right)=G^{-1}\left(\hat{u}\left(k-1\right),\hat{v}\left(k-1\right),c_{12}\left(k-1\right),c_{12}\left(k\right)\right), \end{array}$$

where *G* is the map $G:R^{L+1}\times R^{L+1}\times R^{L+1}\times R^{L+1}\to R^{L+1}$ and, for an arbitrarily fixed $a,b,c\in R^{L+1}$, G(a,b,c,·) is assumed to have an inverse map $G^{-1}\left(a,b,c,\;\cdot \;\right)$. In addition, the following conditions are also assumed to be satisfied:

**(Condition 1:)** For any positive number ε, there exists δ such that if

$$\begin{array}{c} ∥\xi_{1}-\xi_{2}{∥}_{R^{L+1}}<\delta \;\left(\xi_{1},\xi_{2}\in R^{L+1}\right), \end{array}$$

then the following inequality holds:

$$\begin{array}{c} \underset{a,b,c\in R^{L+1}}{\sup}{∥G^{-1}\left(a,b,c,\xi_{1}\right)-G^{-1}\left(a,b,c,\xi_{2}\right)∥}_{R^{L+1}}<\varepsilon . \end{array}$$

Using the synchronization system $\sum_{1}$, after the k=L step, $\hat{u}$ and $\hat{v}$ are synchronized to *u* and *v*, respectively, so that after the same time, under Condition 1, the restored signal $t_{2}\left(k+1\right)$ is synchronized to the transmitted signal $s_{1}\left(k\right)$. If the original image is encrypted to (M+1)×(L+1) pixel image data and is sent from subsystem $S_{1}$ to subsystem $S_{2}$. After the running time needed for the synchronization has passed, the original image data are sliced to $s_{1}\left(k\right)\in R^{L+1}$ (one row at a time), hence the transmission operation should be performed *M* times. At the reception side, the image of (M+1)×(L+1) pixels is obtained by storing the restored data $t_{2}\left(k\right)\in R^{L+1}$ in sequence.

**Remark** **1.** *Although the chaotic neural network in the above has the seven layers and is constructed by learning the van der Pol equation, in the proposed method, any neural network can be used as long as it is chaotic. The information required for decryption is the parameters of the neural network representing the boundary conditions. More specifically, the parameters are the matrices and the bias vectors that represent the linear transformations performed in each layer. If the numbers of input and output variables in a certain layer are $n_{\mathrm{in}}$ and $n_{\mathrm{out}}$, then the number of parameters in this layer is $n_{\mathrm{in}}\times n_{\mathrm{out}}+n_{\mathrm{out}}$. The sum of this number for each layer is the total number of the parameters, which represents the size of the key space. Since the number of the layers and that of the parameters can be changed freely, the size of the key space can be arbitrarily large. However, the larger the neural network becomes, the more difficult it is to be trained. Therefore, there is a trade-off between the security performance and the computational complexity of training.*

**Remark** **2.** *The computational cost of the proposed method is as follows. First, the neural network needs to be trained in advance. The time required for this is difficult to estimate because it depends on the quality of the actual data; however, for example, in the following numerical experiments, the average computation time was 1 min and 48 s for the five trials when using Google Colaboratory and Tesla P100. In addition, this pre-training has to be performed once before encryption and decryption. The time required for encryption is the same as the time required to compute a solution of the wave equation. The solution of the wave equation at each position and time can be obtained in a constant time. Hence, the computational cost is proportional to the image size. However, in reality, the computation is much more efficient than this estimation because the computation of the solution to the wave equation can be parallelized in the spatial direction, and the number of processors, especially in GPUs, typically exceeds the numbers of vertical and horizontal pixels in the image. Therefore, actually, encryption can be expected to be possible within a computation time that is several times shorter for the vertical and horizontal sizes of the image. The same is true for decryption.*

### 2.4. Numerical Experiments

To evaluate the proposed method, numerical experiments were conducted using the neural networks, the training data and the parameters with the following structure.

**Figure 9:**
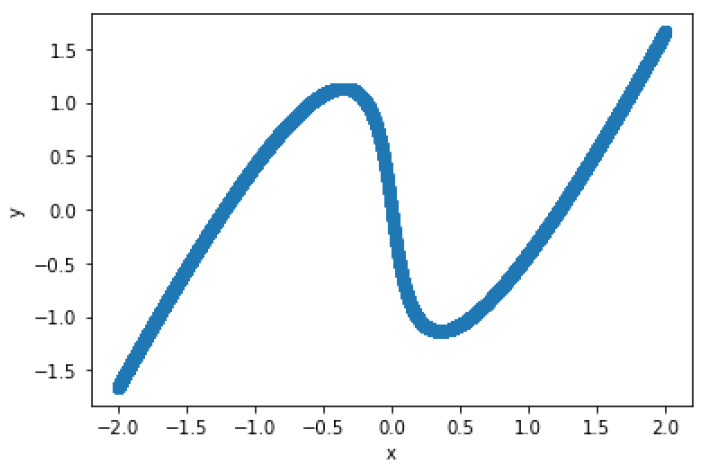
Scatter plot of the relationship between *x* and *y*.

For the training data in the experiment, the input *x* was chosen at equal intervals from the interval (−2,2), and Equation (15),

$$\begin{array}{c} \beta {\left(u-v\right)}^{3}+\left(1-\alpha \right)\left(u-v\right)+2v=0, \end{array}$$

was solved using the Newton method, with the target *y* of the neural network being the solution to the equation. The number of data $N_{d}$ was set to 20,000. The scatter plot of *x* and the solutions *y* for the equation are shown in Figure 9. To reduce the bias of the input data $N=\{x_{1},x_{2},...,x_{N_{d}}\}$ for training, we split the dataset randomly using the train test split function of the Python library Scikit-learn to select the training data and the test data. Here, we use 20% of all data as a test set, so the number of data in the training set is 16,000 and the number of test sets is 4000. The neural network was implemented using PyTorch, and it was executed on a Tesla K80 on Google Colaboratory.

The neural network used in this study was a seven layer perceptron with batch normalization layers (the reasons why the batch normalization layers are used are explained later with data in Table 1 and Table 2).

Each layer has a weight *W* and a bias *b* and performs a nonlinear calculation as follows:

(26) $$\begin{array}{c} \hat{y}=g\left(Wx+b\right). \end{array}$$

**Figure 10:**
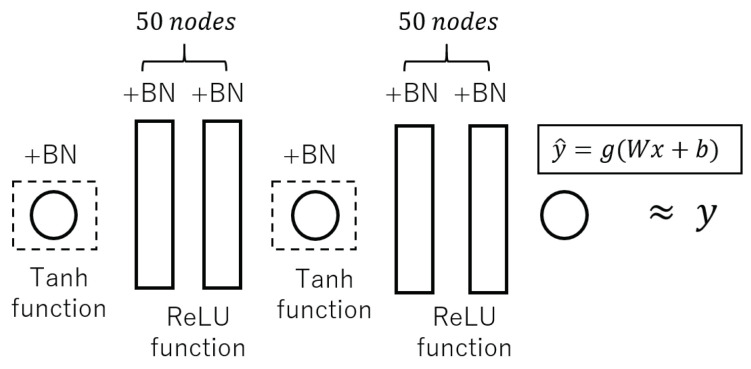
Structure of neural networks in numerical experiments.

As the training depth of the neural network increases, we apply a batch normalization (BN) to each layer of the neural network as shown in Figure 10. This is to keep the overall distribution of each layer from biasing towards the upper and lower limits of the interval of the nonlinear function, which leads to the disappearance of the gradient. BN forces the distribution of the input values to follow a standard normal distribution, making the overall learning process more stable. Therefore, each mini-batch for learning should be normalized. For each mini-batch $B=\{x_{1},x_{2},\cdots ,x_{m}\}$, consisting of *m* data, the average $\mu_{B}$ and variance $\sigma_{B}^{2}$ of the mini-batch are:

(27) $$\begin{array}{cc} \mu_{B} & =\frac{1}{N_{d}}\sum_{i=1}^{N_{d}}x_{i}, \end{array}$$

(28) $$\begin{array}{cc} \sigma_{B}^{2} & =\frac{1}{N_{d}}\sum_{i=1}^{N_{d}}{\left(x_{i}-\mu_{B}\right)}^{2}. \end{array}$$

Batch normalization transforms each data $x_{i}$ in the mini-batch as follows:

(29) $$\begin{array}{cc} {\hat{x}}_{i} & =\frac{x_{i}-\mu_{B}}{\sqrt{\sigma_{B}^{2}+\epsilon }}, \end{array}$$

(30) $$\begin{array}{cc} y_{i} & =\gamma {\hat{x}}_{i}+\beta , \end{array}$$

where γ and β are the parameters of the model so that the output of each layer is normalized. In nonlinear calculations (24), g(·) is the activation function. Since we assumed that the images are grayscale, the first and fourth layers are set as neural network layers with only one node. In these layers, the hyperbolic function (tanh),

(31) $$\begin{array}{c} g\left(r\right)=\tanh\left(r\right)=\frac{e^{r}-e^{-r}}{e^{r}+e^{-r}}, \end{array}$$

is the activation function so that the output converts the input value to a number in the range of −1.0 to 1.0. For layers 2, 3, 5 and 6, 50 nodes were set up and computed using the rectified linear unit (ReLU)

(32) $$\begin{array}{c} g\left(r\right)=\left\{\begin{array}{c} 0,\;for\;r<0\\ r,\;for\;r\geq 0. \end{array}\right. \end{array}$$

**Figure 11:**
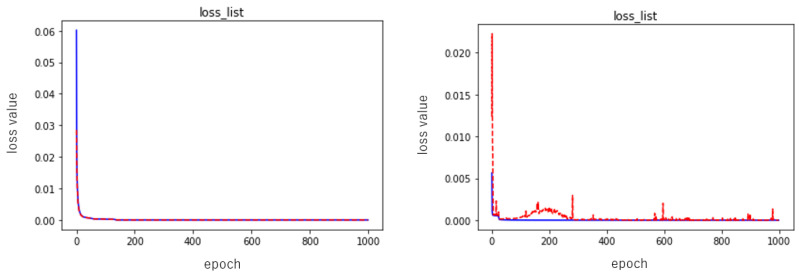
Examples of training error (blue line) and testing error (red line) of the neural network during the learning process. The figure on the left is the result of error training without using BN; the figure on the right is the result of error training using BN.

**Figure 12:**
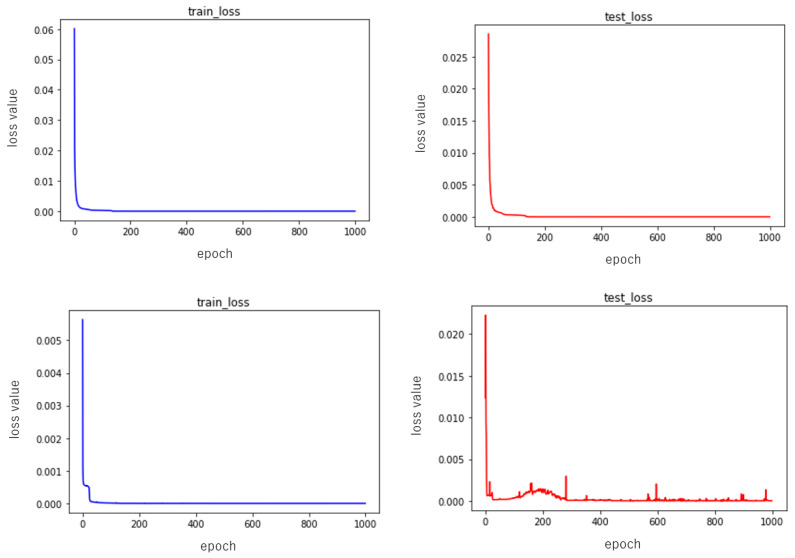
Examples of training error drop and test error drop.The 1st figure is training error without BN; the 2nd figure is testing error without BN; the 3rd figure is training error with BN; the 4th figure is testing error with BN.

Training error, which indicates learning accuracy, and test error, which determines generalization performance, were evaluated using the mean square error (MSE),

(33) $$\begin{array}{c} J=\frac{1}{N}\sum_{i=1}^{N}{\left(y_{i}-{\hat{y}}_{i}\right)}^{2}, \end{array}$$

where ${\hat{y}}_{i}$ is the output value and $y_{i}$ is the target value. We used Adam as the optimization algorithm for parameter updates in training [28]. The learning rate was set to 0.001 and the number of learning epochs was set to 1000. The training and testing errors, for example, are shown in Figure 11 and Figure 12. Since the training results depend on the random numbers used for parameter initialization, we ran the training ten times while changing the seed of the random numbers, and evaluated the performance of the neural network using the mean and standard deviation of the results. Here, we performed two sets of experiments, one in which each layer of the neural network was nonlinearly transformed using the activation function only, and the other in which batch normalization (refer to BN) layers were added to each layer while performing the nonlinear transformation. The performance results of these two groups are shown in Table 1 and Table 2, respectively.

As can be seen from Figure 11, neither the addition of the batch normalization layers nor the absence of the batch normalization layer affects the speed of the descent, and from the given illustration alone, it can also be said that the descent of the experimental group without the batch normalization layers is slightly better. Then, in terms of the values used for evaluation, the training and testing errors of the experimental group without batch normalization layers were 0.000005 ± 0.000001 and 0.000005 ± 0.000001, while the group with batch normalization layers had an error drop of 0.000002 ± 0.000001 and 0.000275 ± 0.000394, so we can say that the neural network with batch normalization layers was slightly better than the one without batch normalization layers. This is one reason why we used batch normalization.

Another important reason is the Lyapunov exponent. Introduced at the beginning of this section, the Lyapunov exponent is a numerical value used to judge whether a system is chaotic. Therefore, we also computed the corresponding Lyapunov exponent for the ten experiments, also taking the mean and standard deviation values for the numerical evaluation. From the results presented in Table 2, the average value of the Lyapunov exponent for the neural network without batch normalization layers is −0.067282, which is less than 0, indicating that this set of trained neural nets does not approximate the chaotic vibrations well. However, the average value of the Lyapunov exponent for the neural network containing the batch normalization layers is 0.024377, which is greater than 0. From this point of view, it can be said that the neural network successfully approximates the boundary conditions, thereby equipping the chaotic behaviors, and hence being suitable for the secret communication system.

**Figure 13:**
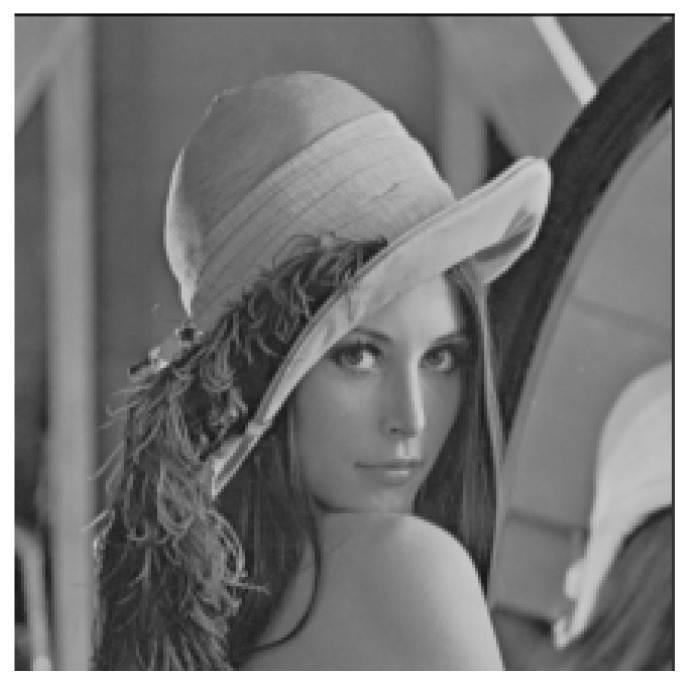
Original grayscale image.

We applied the proposed secret communication system to the image shown in Figure 13 with size 512 × 512 pixels, i.e., M=512,L=512.

**Modulation**

(34) $$\begin{array}{cc} w(k+1)\; & =C\left(w\left(k\right)\right)\{0.03m|u\left(k\right){|}_{V}+0.03m|v\left(k\right){|}_{V}\}+\left\{0.5C\left(w\left(k\right)\right)+0.1I\right\}\\ \; & \times \{0.08m|u\left(k\right){|}_{V}+0.08m|v\left(k\right){|}_{V}+s_{1}\left(k+1\right)\},\\ \; & c_{12}\left(k\right)=w\left(k\right). \end{array}$$

The detailed formulas for modulation and demodulation are shown below, respectively.

**Demodulation**

(35) $$\begin{array}{cc} t_{2}\left(k+1\right)\; & =\frac{1}{m}{\left\{0.5C\left(c_{12}\left(k-1\right)\right)+0.1I\right\}}^{-1}\\ \; & \times \{c_{12}\left(k\right)-C\left(c_{12}\left(k-1\right)\right)\times \left\{0.03m\right|\hat{u}{\left(k-1\right)|}_{V}+0.03m|\hat{v}{\left(k-1\right)|}_{V}\}]\\ \; & -0.08|\hat{u}{\left(k-1\right)|}_{V}-0.08{\left|\hat{v}\left(k-1\right)\right|}_{V}, \end{array}$$

where $w\left(k\right)\in R^{L+1}$, $s_{1}\left(k\right)\in R^{L+1}$, *I* is the (L+1) order unit matrix, and m∈R is the parameter. We also denote $C\left(f\right)=\mathrm{diag}(|f_{0}\left(1-f_{0}\right)|,\cdots ,|f_{L}\left(1-f_{L}\right)|)$ for a vector $f={\left[f_{0},f_{1},\cdots ,f_{L}\right]}^{T}$, and ${\left|f\right|}_{V}=\left[\right|f_{0}\left|,\right|f_{1}\left|,\cdots ,\right|f_{L}{\left|\right]}^{T}$. $s_{1}$ represents the information to be sent. $c_{12}=w$ is the information sent and received between the systems during communication, and $t_{2}$ is the information retrieved by demodulation and corresponds to $s_{1}$ if the two systems are synchronized.

**Figure 14:**
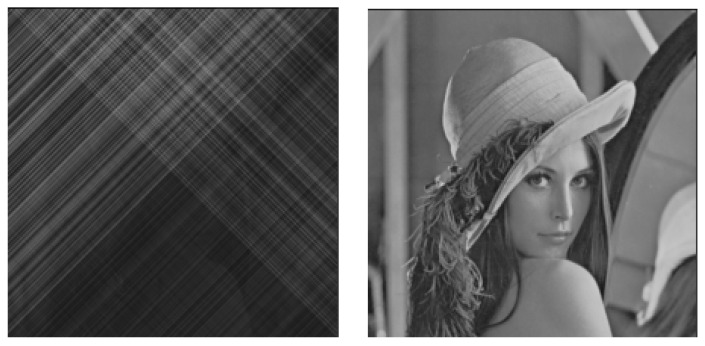
Encrypted grayscale images and restored images in the proposed method of this study. The figure on the left is the encrypted grayscale image; the figure on the right is the restored image.

**Figure 15:**
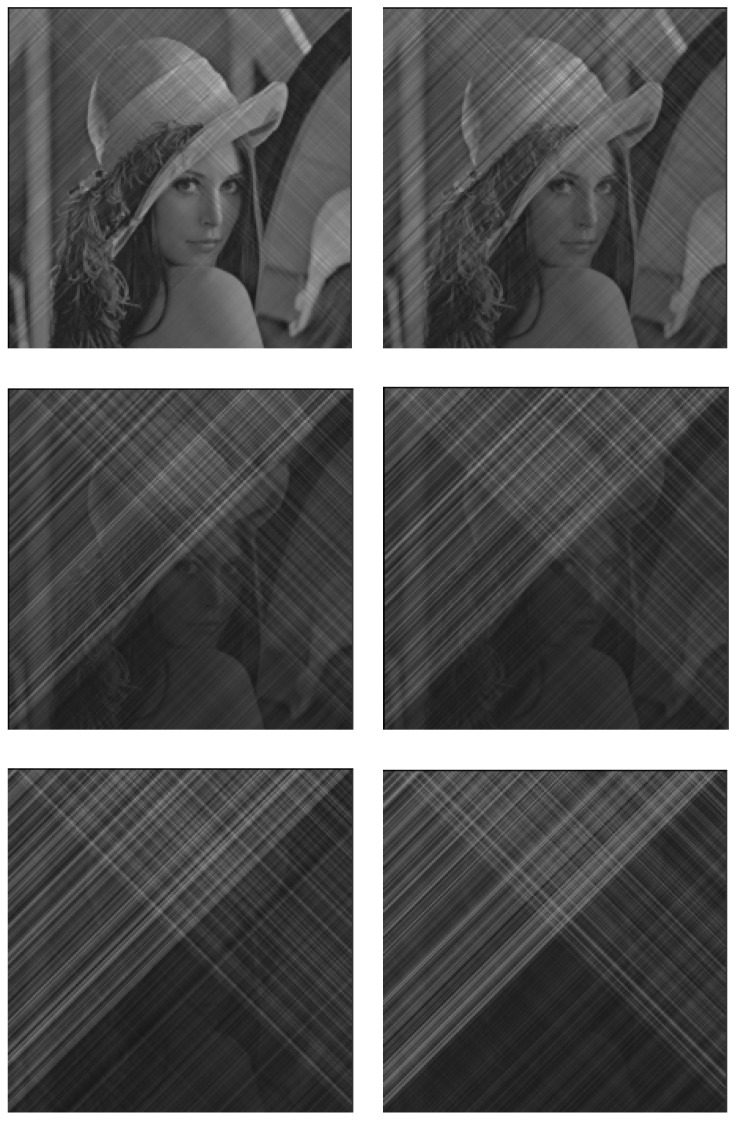
The effect of changing the *m* value in the modulation and demodulation portions on the image encryption effect. Six different values of *m* (*m* = 0.8, 1.25, 2.5, 3.0, 4.5, 6.0) were tried, and the corresponding encryption effects were observed for each, where the larger the value of *m*, the better the secrecy performance of the image, within the computable range.

Figure 14 shows the results of numerical experiments using a system with boundary conditions set by the learned neural network, with the parameters m=6 in the modulation and demodulation sections. The transmitted image is shown in Figure 13. Figure 14 (left) shows the modulated image after passing through the secret communication system, and Figure 14 (right) shows the image recovered by the synchronous system. In addition, m=6 is a parameter value obtained after numerous attempts, and the size of *m* affects the outcome of the entire modulation and demodulation operations (see Figure 15).

As shown in Figure 13, the original image is almost unrecognizable from the encrypted image. Figure 14 (right) shows the reconstructed image after the demodulation section. There is little distinction between the transmitted image and the reconstructed image.

## 3. Numerical Experiments with Color Images

In the previous section, we explained the wave equations with van der Pol boundary conditions and the corresponding synchronization systems. We then improved the security of the confidential communication system by approximating the chaotic phenomena with a neural network; however, relatively simple grayscale images are used as input objects. Next, we will replace the input images with colored ones and conduct experiments to see if the proposed approach is still applicable to those images. The biggest difference between a color image and a grayscle image is that each pixel of a color image is usually represented by three components, red (R), green (G) and blue (B), of which intensities are represented by numerics between, for example, (0, 255), while a pixel of a grayscale image has a single component. This results in three differences: one being the structure of the neural network itself, the second being that the training method for each pixel is also optional, which means the three components (R, G and B) of each pixel can be trained separately by themselves or mixed together, and the third being the Lyapunov exponent for testing chaotic phenomena.

Color images in the neural network can be computed, one by one, as

(36) $$\begin{array}{c} x_{r}\to f\left(x_{r}\right) \end{array}$$

(37) $$\begin{array}{c} x_{g}\to f\left(x_{g}\right) \end{array}$$

(38) $$\begin{array}{c} x_{b}\to f\left(x_{b}\right). \end{array}$$

The neural network structure in this approach is still one input neuron and one output neuron; they are represented by (36)–(38) and are trained separately. However, the security of this approach is not very high and there remains a risk of theft. Therefore, in this study, after R, G and B are fed into the neural network, we will mix and disrupt them, adopting a structure that has three input neurons and three output neurons, thus improving the security of the confidential communication system.

**Figure 16:**
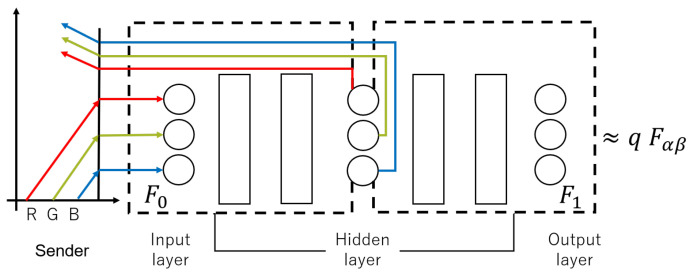
Relationship between the structure of Neural Networks and boundary conditions with RGB.

The composition diagram of applying RGB to the neural network is shown in Figure 16, where the data from three neurons are used as inputs to the input layer and then sent to the hidden layer. First, since color images are involved, $\sum_{0}$ and $\sum_{1}$ correspond to each color in RGB, and the dependent variables *u* and *v* are expanded to three dimensions. The *u* and *v* are then discretized according to the upwind difference method to obtain the values of u,v at the next time step. (For this part of the process we can refer to Figure 5 in Section 2.3). Because the number of inputs is increased from one to three, the number of the nodes of the first, middle and the last layers are also increased accordingly. By using the input image, the $x_{r}$, $x_{g}$, and $x_{b}$ are computed by using chaotic maps as follows:

(39) $$\begin{array}{cc} \; & x_{r}↘\\ \; & x_{g}\to g_{i}\left(x_{r},x_{g},x_{b}\right)\to f\left(g_{i}\left(x_{r},x_{g},x_{b}\right)\right)\;\left(i=1,2,\cdots ,h_{n}\right),\\ \; & x_{b}↗ \end{array}$$

where $h_{n}$ is the number of neurons in the hidden layer. The former half of the neural network, $F_{0}$, is treated as the left boundary condition, and the latter half, $F_{1}$, is treated as the right boundary condition:

(40) $$\begin{array}{c} u\left(1,t\right)=F_{1}\left(v\left(1,t\right)\right)\\ v\left(0,t\right)=F_{0}\left(u\left(0,t\right)\right). \end{array}$$

We know that the existence of chaos can be determined by calculating the Lyapunov exponent of the dynamical system. If the images are grayscale, the Lyapunov exponent has just one value, hence whether the system is chaotic or not can be determined by examining the exponent. When confronted with color images, the Lyapunov exponent is no longer 1-dimensional due to dimensional growth. k-dimensional space has *k* Lyapunov exponents. In fact, the Lyapunov exponent $\left\{\lambda_{1},\lambda_{2},\cdots ,\lambda_{k}\right\}$ is defined as

(41) $$\begin{array}{c} \left(e,e^{\lambda_{1}},e^{\lambda_{2}},\cdots ,e^{\lambda_{k}}\right)=\lim_{N\to \infty }\left[\mathrm{magnitude}\;\mathrm{of}\;\mathrm{the}\;\mathrm{eigenvalues}\;\mathrm{of}\prod_{n=0}^{N-1}J{\left(x_{n}\right)}^{1/N}\right], \end{array}$$

and

(42) $$\begin{array}{c} J\left(x_{n}\right)=\left(\frac{\partial G_{i}}{\partial x_{i}}\right) \end{array}$$

is the Jacobian matrix of the map $G(x_{n})$. Normally, if the maximum Lyapunov exponent $\lambda_{1}$ is positive, the system can be regarded as chaos, but even if $\lambda_{1}<0$, there may be a latent chaotic case which cannot be observed [27]. In this study, as long as one of the Lyapunov exponents is positive, we conclude that the neural network contains chaotic vibrations, which means that the approximation of the chaotic boundary condition is successful.

For the numerical experiments, we first trained the neural networks so that the chaotic boundary condition is approximated. We choose training data, from which input data are sampled from (−2, 2). Since a pixel has three components, the total amount of data should preferably be a multiple of 3; we choose $N_{d}=$ 30,000 and (15) is again solved by the Newton method. The data are randomly divided into a training set and a test set in the ratio of 8:2. The neural network was implemented using PyTorch, and it was executed on a Tesla T4 from Google Colaboratory.

The structure of the neural network is still a seven layer multilayer perceptron with three neurons in the first, fourth and seventh layers, and 50 neurons in the other hidden layers. Each layer is nonlinearly transformed by $\hat{y}=g\left(Wx+b\right)$ and accompanied by BN, where the activation functions of the first and fourth layers are tanh functions and the others are ReLU functions. The mean squared error is still used for the error function. The entire training process was updated with the Adam algorithm with a learning rate of 0.001. The training was completed after 1000 training runs.

**Figure 17:**
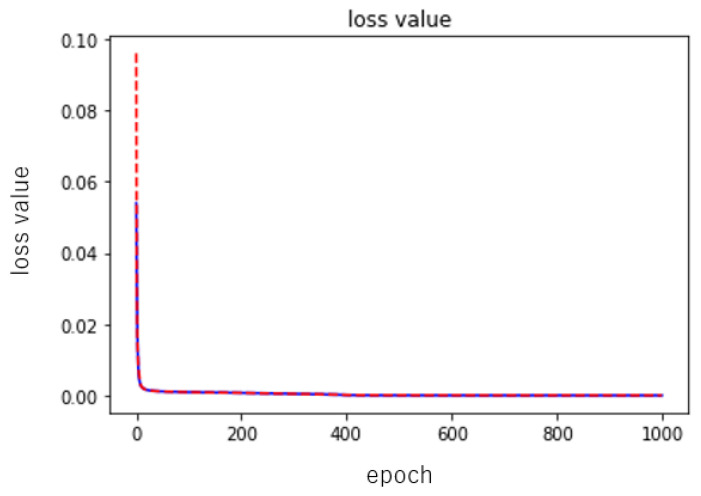
Examples of training error (blue line) and testing error (red line) of the neural network during the learning process.

Table 3 shows the numerical evaluation of the training results and the test results of the neural network. A total of ten experiments were conducted, and each time the seed value was changed so that the initial values of the parameters of the neural networks are changed. The mean and standard deviation are computed for the experimental results. We can see that the results of training and testing are 0.000694 ± 0.000035 and 0.000919 ± 0.000033, respectively, and Figure 17 shows the error decrease of one of the trainings, from which we can clearly see that the training error and the testing error both decrease rapidly, and there is almost no error rebound. Then, we calculated the Lyapunov exponent, respectively, $\lambda_{1}=0.1144\pm 0.1469,\lambda_{2}=0.0038\pm 0.0917$ and $\lambda_{3}=-0.1717\pm 0.2333$, where $\lambda_{1}$ and $\lambda_{2}$ are positive, so we can conclude that the neural networks are in fact chaotic, which somehow approximates the van der Pol boundary condition.

**Figure 18:**
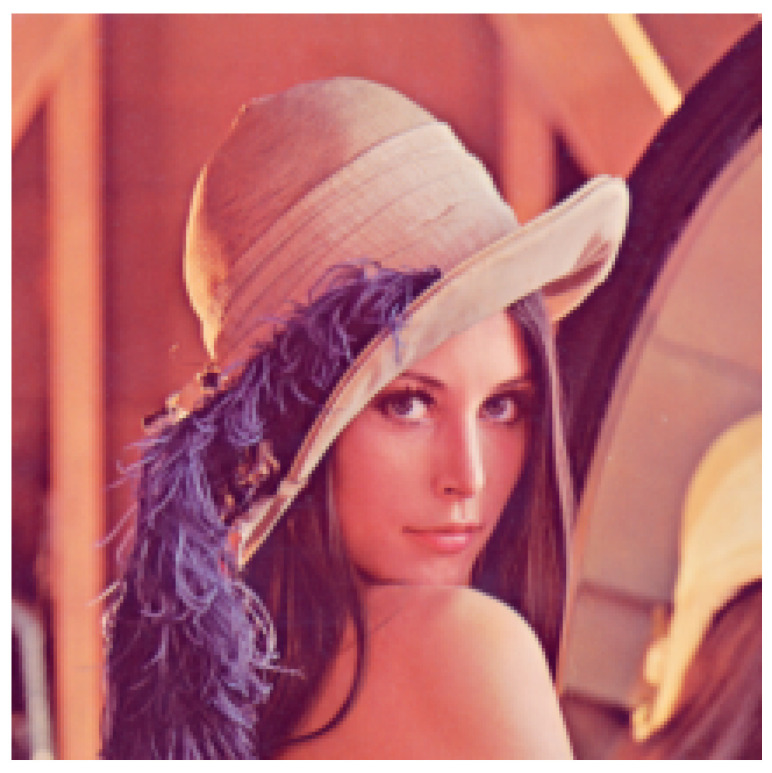
Original color image.

A color image of size 512 × 512, as shown in Figure 18, is put into the whole system as the input. The modulation and demodulation are shown below.

**Modulation**

(43) $$\begin{array}{cc} w(k+1)\; & =C\left(w\left(k\right)\right)\{0.03m|u\left(k\right){|}_{V}+0.03m|v\left(k\right){|}_{V}\}+\left\{0.5C\left(w\left(k\right)\right)+0.1I\right\}\\ \; & \times \{0.08m|u\left(k\right){|}_{V}+0.08m|v\left(k\right){|}_{V}+s_{1}\left(k+1\right)\},\\ \; & c_{12}\left(k\right)=w\left(k\right). \end{array}$$

**Demodulation**

(44) $$\begin{array}{cc} t_{2}\left(k+1\right)\; & =\frac{1}{m}{\left\{0.5C\left(c_{12}\left(k-1\right)\right)+0.1I\right\}}^{-1}\\ \; & \times \{c_{12}\left(k\right)-C\left(c_{12}\left(k-1\right)\right)\times \left\{0.03m\right|\hat{u}{\left(k-1\right)|}_{V}+0.03m|\hat{v}{\left(k-1\right)|}_{V}\}]\\ \; & -0.08|\hat{u}{\left(k-1\right)|}_{V}-0.08{\left|\hat{v}\left(k-1\right)\right|}_{V}. \end{array}$$

**Figure 19:**
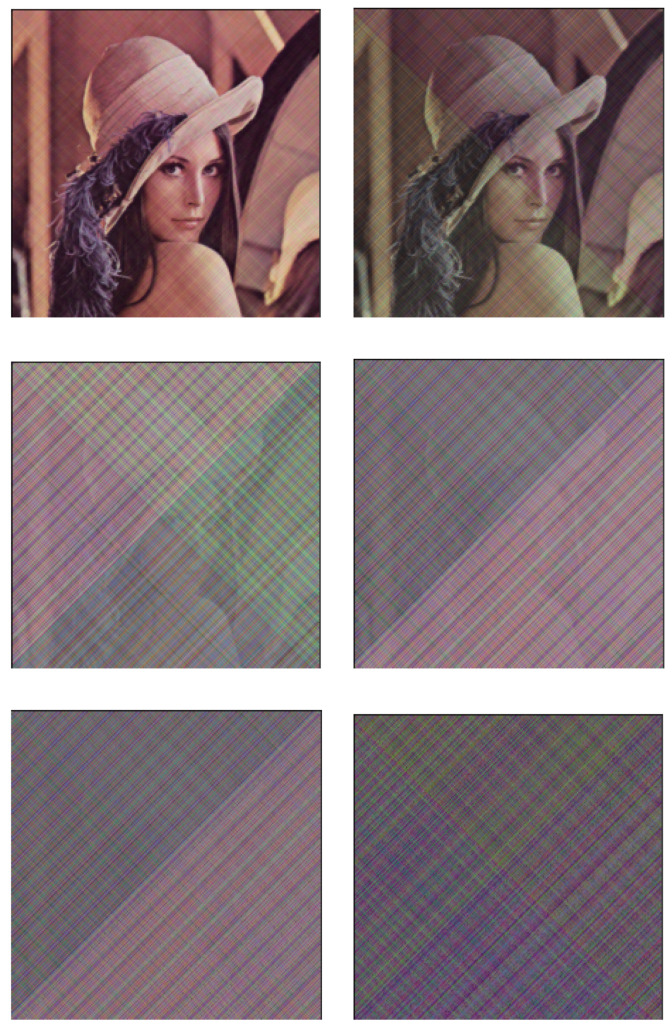
The effect of changing the *m* value in the modulation and demodulation portions on the image encryption effect. Six different values of *m* (*m* = 1.0, 2.0, 5.0, 6.0, 7.5, 8.8) were tried, and the corresponding encryption effects were observed for each, where the larger the value of *m*, the better the secrecy performance of the image, within the computable range.

**Figure 20:**
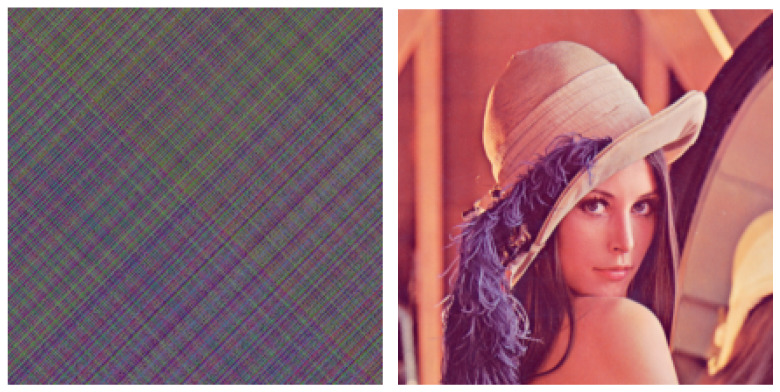
Encrypted color image and restored image in the proposed method of this study. The figure on the left is the encrypted color image; the figure on the right is the restored color image.

With (43) and (44), we can modulate and recover the images; however, the parameter *m* still needs to be tried. In Figure 19, we have tried six different values of *m*. It can be seen that the greater the value of *m*, the better the secrecy of the image. Figure 18 shows the experimental results when the value of m=8.8. Figure 20 (left) is the image that has been secreted by the proposed system, from which we can conclude that the proposed method is successfully applied to the transmission of color images. In fact, it is very difficult to know the original image from the transmitted one while the restored image shown in Figure 20 (right) is almost identical to the original color image.

## 4. Security Evaluation of the Encrypted Images by the Proposed Method

In the previous section, we numerically examined the proposed method in which the chaotic synchronization system and the artificial neural network are combined. In particular, we tested different values of *m* to observe the efficiency of the encryption and decryption of the images. In this section, we test the security of the encrypted images. For some different images and the values of *m*, we investigate whether the encrypted images are secure or not by computing several numerical security measures and color histograms.

Based on traditional evaluation ideas, we first test the randomness of encrypted images: the correlation coefficient and the UACI value [29]. We compare the proposed method with Advanced Encryption Standard (AES) as a reference method. AES is a traditional encryption method, which is a group cipher, where the plaintext is divided into groups of equal length and each group is encrypted until the entire plaintext is encrypted. We used AES in the ECB mode.

Although these are standard measures of security, note that the above measures are not suitable for the proposed approach. In fact, in order to choose suitable measures, the term of “ideal encrypted image” needs to be specified [30]. In typical statistical measures, encrypted images are considered to be secure for, for example, differential attacks when it exhibits randomness. On the other hand, the proposed approach is not designed to generate pseudo-random sequences because a different criterion is used for the term “ideal encrypted image”. The proposed approach aims to encrypt the images into almost identical wave images, which are considered to be “ideal” in this study. The idea behind this is that because, for example, the differential attacks essentially try to find how differences in the original images affect the encrypted images, if the encrypted images are almost identical, then the attacks should fail and the information on the original image cannot be retrieved from them.

Although the statistical measures are not necessarily suitable for the proposed method, to a certain extent, the method has a good statistical property as shown below. In addition, we also performed more appropriate tests, in which the distinguishability of two encrypted images is checked.

### 4.1. Randomness Testing of Encrypted Images

Firstly, we computed the correlation coefficients between adjacent pixels for the encrypted image in three different directions: horizontal, vertical and diagonal. Suppose that the image has N×N pixels $(x_{i},y_{j}),\;i=1,\ldots ,N,\;j=1,\ldots ,N$. We calculate the correlation coefficients for R, G and B values, respectively:

(45) $$\begin{array}{c} r_{xy}=\frac{\frac{1}{N}\sum_{i=1}^{N}\sum_{j}^{N}\left(x_{i}-E\left(x\right)\right)\times \left(y_{i}-E\left(y\right)\right)}{\sqrt{\frac{1}{N}\sum_{i=1}^{N}{\left(x_{i}-E\left(x\right)\right)}^{2}}\sqrt{\frac{1}{N}\sum_{j=1}^{N}{\left(y_{j}-E\left(y\right)\right)}^{2}}}, \end{array}$$

where $E\left(x\right)=\frac{1}{N}\sum_{i}^{N}x_{i},E\left(y\right)=\frac{1}{N}\sum_{j}^{N}y_{j}$. The correlation coefficients are between −1 and 1. For example, when the value is close to 1, it means a high correlation between pixels. When it is close to 0, there is no correlation; hence the image is considered pseudo-random. The results are shown in Table 4 for the grayscale images and in Table 5 for the color images. The images encrypted by the proposed method all present a high inter-pixel correlation, while the traditional AES encryption has an ideal value of around 0. This is because the encrypted images of the proposed method have a certain regularity that is caused by the fact that the images represent the trajectories of waves. Note that this regularity does not imply the recognizability of the original images.

Secondly, we computed UACI values, which measure how much the encrypted image differs from the original image. For two different images, $I_{1}$ and $I_{2}$, of the same size N×N, the UACI values between them is defined by:

(46) $$\begin{array}{c} UACI=\frac{1}{N^{2}}\sum_{i=1}^{N}\sum_{j=1}^{N}\frac{I_{1}\left(i,j\right)-I_{2}\left(i,j\right)}{\mathrm{tonal}\;\mathrm{range}}\times 100\%. \end{array}$$

Considering the color range and gray value distribution of images, the ideal value is found to be 33.3. The closer the UACI of the encrypted image is to this ideal value, the better the security.

The computed values are shown in Table 4 for the grayscale images and in Table 6 for the color images. All the results for AES are about 50.0, while those for the proposed method depend on the target images. AES performs better for the images of vegetables; however, the values of the proposed method for the images of the woman are better than those of AES.

Thirdly, we observed the histograms, which are graphical representations of the intensity distribution of pixels in an image. For gray images, the grayscale histogram reflects the grayscale statistical information of the image. For example, each grayscale image of the previous numerical tests has 256 intensity levels with values from 0 to 255. We store the number of pixels corresponding to each gray level in a 256 capacity array. Similarly, for color images, we can compute the histograms for each of the three different channels, R, G and B, respectively.

**Figure 21:**
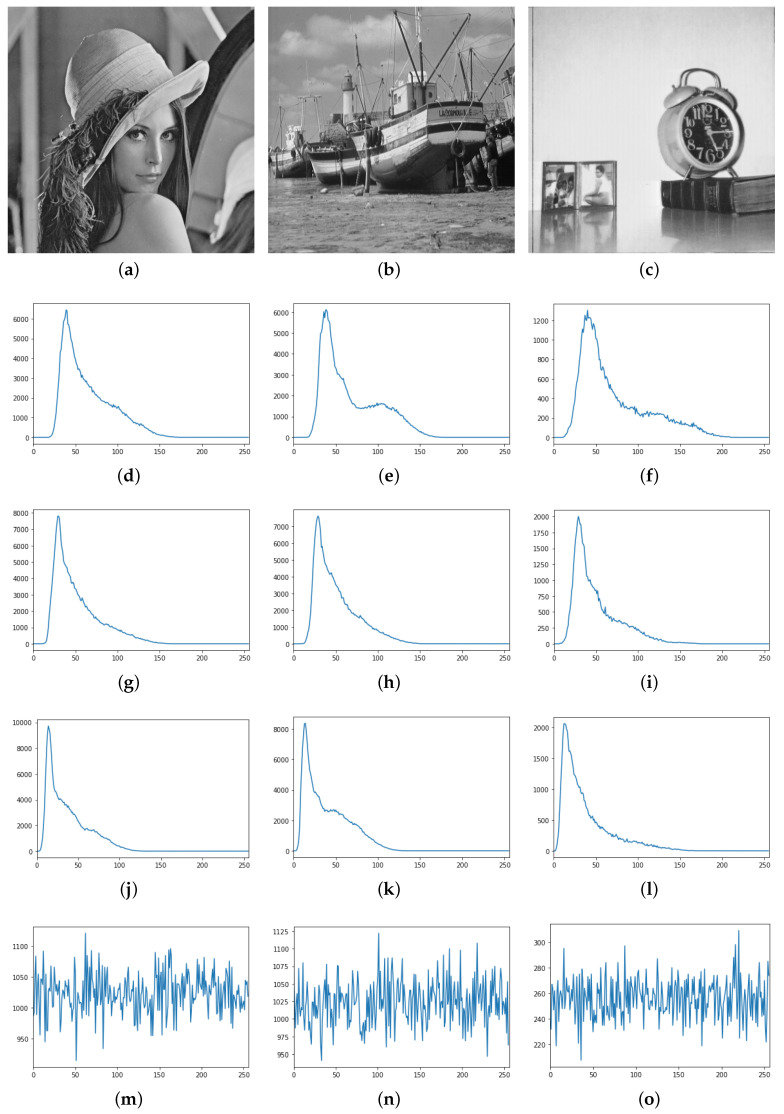
The grayscale images and the histograms of the encrypted images. The first row shows three different grayscale images (**a**–**c**). In the second, third and fourth rows, the three figures in each row represent the histograms of the grayscale values of the three original images encrypted by the proposed approach under the settings of T=1,2,3 and m=6.0, respectively (**d**–**l**). The last row shows the histogram after encryption by the AES. The horizontal coordinate represents the tonal range and the vertical coordinate represents the absolute frequency (**m**–**o**).

**Figure 22:**
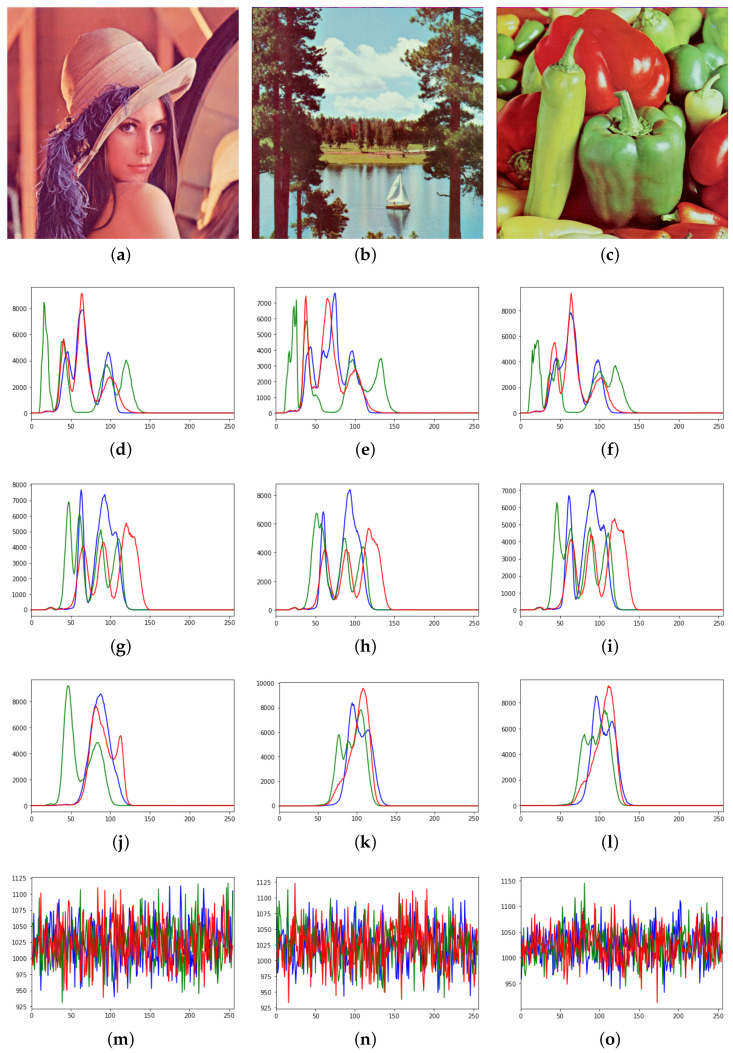
The color images and the histogram of the encrypted image. The first row shows three different color images (**a**–**c**). In the second, third and fourth rows, the three figures in each row represent the histograms of the three original images encrypted by the proposed approach under the settings of T=1,2,3 and m=6.0, respectively. The last row shows the histogram after encryption by the AES. The red, green and blue colors correspond to the histograms of each channel of R, G and B, respectively (**d**–**l**). The last row shows the histograms after encryption by the AES. The horizontal coordinates represent the tonal range and the vertical coordinates represent the absolute frequency (**m**–**o**).

The results are shown in Figure 21 for the grayscale images and in Figure 22 for the color images. We can see that the histograms of the images encrypted by AES show a uniform distribution, both in color and grayscale, which to some extent indicates that the encrypted images are disordered and hence in a secure state.

**Figure 23:**
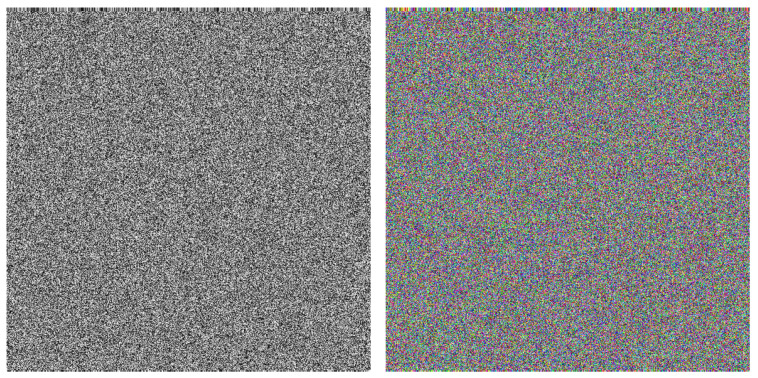
The grayscale encrypted image (**left**) and the color encrypted image (**right**) obtained by using the AES on the grayscale image (Figure 13) and the color image (Figure 20).

Meanwhile, the histogram of the encrypted image in the mode of the proposed method presents an unbalanced state with a high concentration in a certain region. The reason for this phenomenon is as follows. The proposal method uses the reflection of waves, and even though the initial conditions are random values, they are propagated to the left and right sides along straight lines. In fact, as shown in Figure 23, the images encrypted by AES and the images encrypted by the proposed method are apparently different from each other. The former has almost no structure, and the latter a visible structure that corresponds to the trajectories of the waves. The above mentioned concentration is observed because the intensity of a trajectory takes the same value.

From this consideration, we deduce that the difference in the position of the highest point of the histogram reflects the difference in the lengths of the straight lines, which in turn depend on the waiting time for synchronization. To confirm this, we computed the histograms changing the waiting time as T×(theverticalsizeofthetargetimage) with T=1,2,3. For grayscale images, the highest point of the histogram increases with the waiting time, and for color images, R, G and B have multiple peaks and the position and the number of the peaks depend on the waiting time. This implies that although the histograms of the encrypted images have some peaks, from these peaks only the waiting time for synchronization can be estimated, and at least in a naive way, no information on the original images can.

### 4.2. Distinguishability of Encrypted Images

From the above security tests, we can say that AES has higher security from the perspective of randomness; however, the AES and the proposed approach are fundamentally different in the definition of “ideal encrypted images”. Hence, it is important to investigate the security issue from another perspective.

Since the ideal encrypted images of the proposed approach are “the indistinguishable images of waves”, we calculated the similarity of encrypted images of two different images. If there is a high correlation between the two, it indicates that the proposed method encrypts different images into almost identical encryption results. In addition, if two encrypted images have high similarity, there is a very low probability of leaking the original image information.

We evaluated three inter-image-similarity criteria [31,32] for color and grayscale images. Firstly we use the averaging pixel values,

(47) $$\begin{array}{cc} \; & \frac{\sqrt{{\left(\bar{I1_{R}}-\bar{I2_{R}}\right)}^{2}+{\left(\bar{I1_{G}}-\bar{I2_{G}}\right)}^{2}+{\left(\bar{I1_{B}}-\bar{I2_{B}}\right)}^{2}}}{\sqrt{3\times {\left(255-0\right)}^{2}}}, \end{array}$$

where I1,I2 are images and the averaging value, for example, $\bar{I_{R}}$, for the image *I* is

$$\begin{array}{c} \bar{I_{R}}=\frac{1}{N_{\mathrm{pixel}}}\sum_{i=0}^{N_{\mathrm{pixel}}}I_{R_{i}},I_{R}=\left\{I_{R_{0}},I_{R_{1}},\ldots ,I_{R_{N_{\mathrm{pixel}}}}\right\}. \end{array}$$

$N_{\mathrm{pixel}}$ is the number of pixels and $I_{R_{i}}$ is the red value of the *i*th pixel of the image *I*. Secondly, we use the histogram for pixel values,

(48) $$\begin{array}{cc} \; & \frac{\sqrt{\sum_{i=0}^{N_{\mathrm{int}}}{\left(I1_{RH_{i}}-I2_{RH_{i}}\right)}^{2}}+\sqrt{\sum_{i=0}^{N_{\mathrm{int}}}{\left(I1_{GH_{i}}-I2_{GH_{i}}\right)}^{2}}+\sqrt{\sum_{i=0}^{N_{\mathrm{int}}}{\left(I1_{BH_{i}}-I2_{BH_{i}}\right)}^{2}}}{3\times \sqrt{2\times {\left(1-0\right)}^{2}}},\\ \; & I_{RH}=\left\{I_{RH_{0}},I_{RH_{1}},\cdots ,I_{RH_{N_{\mathrm{int}}}}\right\}, \end{array}$$

where I1,I2 are images and $N_{\mathrm{int}}$ is the number of the levels of intensity, and $I_{RH_{i}}$ is the relative degree of the *i*th bin of the histogram of the red values of the image *I*. Thirdly, we use the correlation coefficients:

(49) $$\begin{array}{c} 1-\left|\frac{r_{I1I2_{R}}+r_{I1I2_{G}}+r_{I1I2_{B}}}{3}\right|, \end{array}$$

where

$$\begin{array}{c} r_{I1I2_{R}}=\frac{\sum_{i=0}^{N_{\mathrm{pixel}}}\left(I1_{R_{i}}-\bar{I1_{R}}\right)\left(I2_{R_{i}}-\bar{I2_{R}}\right)}{\sum_{i=0}^{N_{\mathrm{pixel}}}{\left(I1_{R_{i}}-\bar{I1_{R}}\right)}^{2}\sum_{i=0}^{N_{\mathrm{pixel}}}{\left(I2_{R_{i}}-\bar{I2_{R}}\right)}^{2}}. \end{array}$$

In the experiments, the resolutions of the two images are unified into the same value. We computed these values changing the value of *m* and the waiting time for synchronization.

From the results of the grayscale and color images shown in Table 7 and Table 8, it can be seen that the correlation coefficients between the encrypted images of different images are significantly large. The values are indeed almost close to one; when ten decimal places are used, the values of correlation coefficients are, for example, 0.9999999977 for the grayscale image of “boat” with m=7.5,T=1 and that of “lena” with m=6.0,T=1 and 0.9999999865 for the color image of “boat” with m=7.5,T=1 and that of “lena” with m=8.8,T=2. The other two measures are very small in all cases, indicating high indistinguishability between the encrypted images, hence the high security of the proposed approach.

## 5. Conclusions

The purpose of this study is to establish a more stable and secure communication system by improving the method reported in [19]. The reason for this is that the number of parameters in a confidential communication system, using the chaotic synchronization system from [19], is too small, and the image information can be easily stolen so that even if only one parameter is attacked, there is a certain chance that the original image can be seen in its entirety. In order to improve the security of the system based on the original concept, the development of a new method that includes a chaotic system with a larger number of independent parameters is necessary. Therefore, this study proposes a confidential communication system for color image communication using a chaotic synchronous distribution system and deep learning. Deep neural networks have properties that satisfy both conditions; on the one hand, they can approximate any function, and on the other hand, they have a complicated structure so that it is difficult to steal all the information. Therefore, we use a neural network to approximate the van der Pol boundary condition, so that the learned neural network can exhibit chaotic behavior instead of the original boundary condition. In the construction of the neural network, we consider the middle layer of the hidden layer as an output layer as well, and the number of neurons in the input and output layers is related to the input image, so that the network can be divided into two parts, corresponding to the left and right boundary conditions, respectively.

We first used grayscale images as experimental objects to test whether the proposed approach is practically applicable. The neural network has one input neuron and one output neuron, and the intermediate layer is also set to one neuron, and two different activation functions, Tanh and ReLU, are used for nonlinear transformation. The experimental results are analyzed from two aspects: first, during the training and testing of the neural network itself, the error decreases very quickly and there is no error rebound, and the value of the Lyapunov exponent is also positive, which shows that the neural network can learn the van der Pol boundary conditions well to obtain the chaotic phenomenon; second, the modulated image can well mask the information of the original grayscale image, and the recovered image is the same as the original grayscale image. After confirming that the proposed approach can be applied to simpler grayscale images, we further experimented with color images. Since the pixels are expanded from one dimension to three dimensions, a three-input, three-output neural network structure is adopted for a better stealth effect. The experimental results are very close to those of the grayscale images, the neural network still learns the chaotic phenomena, and it can hide the images and restore the images well.

Several security tests were also performed. The proposed method is not designed to generate pseudo-random sequences as the encrypted images. Therefore, statistical tests were not appropriate, and in many cases AES produced images that were closer to random numbers. However, in terms of the UACI value, the proposed method was superior in some cases. On the other hand, if the encrypted images are identical regardless of the original image, then it becomes difficult to infer the original image from the encrypted image. We performed some tests from this perspective as well. The results imply that the encrypted images from the proposed method are almost the same regardless of the original images, thereby showing that the proposed method is certainly secure.

Although we have confirmed to some extent that the techniques used in this study can be applied to color images, there is still room for improvement. In particular, the neural network in this study learns chaotic phenomena by approximating $qF_{\alpha ,\beta }$, which is still somewhat risky. In the future, instead of approximating a function, new activation functions will be created to make the network behave in a chaotic manner.

In conclusion, it is clear from the research conducted so far that the proposed technique can be used in a secure communication system for images, and that it has been effective and has further increased the difficulty of theft. However, the need to improve on the details to make the technique more perfect is definitely a future issue.

## Figures and Tables

**Table 1:** Mean ± standard deviation of results performed 10 times without BN.

Epoch	Data Set	Mean ± Standard
1000 times	train set	0.000005 ± 0.000001
	test set	0.000005 ± 0.000001
Lyapunov exponent	−0.067282 ± 0.102745	

**Table 2:** Mean ± standard deviation of results performed 10 times with BN.

Epoch	Data Set	Mean ± Standard
1000 times	train set	0.000002 ± 0.000001
	test set	0.000275 ± 0.000394
Lyapunov exponent	0.024377 ± 0.148675	

**Table 3:** Mean ± standard deviation of results performed at 10 times with RGB.

Epoch	Data Set	Mean ± Standard
1000 times	train set	0.000694 ± 0.000035
	test set	0.000919 ± 0.000033
Lyapunov exponent	0.1144 ± 0.1469	
	0.0038 ± 0.0917	
	−0.1717 ± 0.2333	

**Table 4:** Correlation test in horizontal, vertical and diagonal directions and UACI test for different grayscale images. Different *m* (m=6,7.5) and *T* (T=1,2,3) were tried respectively, where “lena” is Figure 21a, “boat” is Figure 21b, and “clock” is Figure 21c.

Object			Correlation			UACI
			Horizontal	Vertical	Diagonal	
lena	*m* = 6	*T* = 1	0.972642	0.972993	0.922492	36.141486
		*T* = 2	0.962627	0.962568	0.890740	39.410799
		*T* = 3	0.968880	0.969089	0.915038	35.114740
	*m* = 7.5	*T* = 1	0.968823	0.969244	0.908995	39.877836
		*T* = 2	0.954396	0.954471	0.874803	39.730604
		*T* = 3	0.976311	0.976517	0.940879	35.622222
	AES		0.002630	0.008785	0.000658	49.996347
boat	*m* = 6	*T* = 1	0.980538	0.980823	0.943327	36.728787
		*T* = 2	0.961264	0.961193	0.885866	41.783973
		*T* = 3	0.973791	0.973994	0.930897	39.928194
	*m* = 7.5	*T* = 1	0.972897	0.973300	0.923706	36.761638
		*T* = 2	0.957479	0.957473	0.874213	41.551513
		*T* = 3	0.973904	0.974118	0.936444	40.359627
	AES		0.000163	0.000445	0.000502	50.000055
clock	*m* = 6	*T* = 1	0.921742	0.922687	0.806450	48.089881
		*T* = 2	0.858309	0.858372	0.664064	58.067992
		*T* = 3	0.888560	0.889011	0.722784	58.823428
	*m* = 7.5	*T* = 1	0.899274	0.900966	0.770316	47.179087
		*T* = 2	0.853312	0.957473	0.874213	56.637741
		*T* = 3	0.880133	0.881703	0.754349	57.388910
	AES		0.005367	0.004363	0.003360	49.929277

**Table 5:** Correlation test in horizontal, vertical and diagonal directions with different color images. Different values of *m* (m=7.5,8.8) and *T* (T=1,2,3) were tried respectively, where “lena” is Figure 22a, “boat” is Figure 22b, and “veg” is Figure 22c.

Object			Correlation								
			Horizontal			Vertical			Diagonal		
			R	G	B	R	G	B	R	G	B
lena	*m* = 7.5	*T* = 1	0.97	0.99	0.98	0.97	0.99	0.98	0.95	0.99	0.96
		*T* = 2	0.99	0.99	0.98	0.99	0.99	0.98	0.98	0.98	0.97
		*T* = 3	0.93	0.94	0.81	0.93	0.94	0.81	0.82	0.89	0.59
	*m* = 8.8	*T* = 1	0.96	0.99	0.97	0.96	0.99	0.97	0.88	0.88	0.88
		*T* = 2	0.98	0.99	0.98	0.98	0.99	0.97	0.96	0.98	0.96
		*T* = 3	0.89	0.95	0.82	0.90	0.95	0.83	0.75	0.88	0.57
	AES		5 ×$10^{-3}$	1 ×$10^{-3}$	−1 ×$10^{-3}$	1 ×$10^{-2}$	6 ×$10^{-3}$	8 ×$10^{-3}$	1 ×$10^{-3}$	−8 ×$10^{-5}$	3 ×$10^{-3}$
boat	*m* = 7.5	*T* = 1	0.96	1.00	0.98	0.97	1.00	0.98	0.94	0.99	0.96
		*T* = 2	0.99	0.99	0.98	0.99	0.99	0.98	0.98	0.98	0.98
		*T* = 3	0.92	0.97	0.92	0.93	0.97	0.92	0.82	0.94	0.86
	*m* = 8.8	*T* = 1	0.96	0.99	0.97	0.96	0.99	0.97	0.93	0.99	0.95
		*T* = 2	0.98	0.99	0.97	0.98	0.99	0.97	0.96	0.98	0.96
		*T* = 3	0.92	0.96	0.92	0.93	0.97	0.93	0.82	0.93	0.87
	AES		6 ×$10^{-4}$	−1 ×$10^{-3}$	−1 ×$10^{-4}$	−3 ×$10^{-3}$	−1 ×$10^{-3}$	−3 ×$10^{-3}$	1 ×$10^{-3}$	−2 ×$10^{-3}$	−3 ×$10^{-3}$
veg	*m* = 7.5	*T* = 1	0.97	0.99	0.98	0.97	0.99	0.98	0.94	0.99	0.96
		*T* = 2	0.99	0.99	0.98	0.99	0.99	0.88	0.98	0.98	0.97
		*T* = 3	0.92	0.97	0.92	0.93	0.97	0.92	0.82	0.94	0.86
	*m* = 8.8	*T* = 1	0.96	0.99	0.97	0.97	0.99	0.98	0.94	0.99	0.96
		*T* = 2	0.98	0.99	0.97	0.98	0.99	0.97	0.96	0.98	0.96
		*T* = 3	0.92	0.96	0.92	0.93	0.96	0.92	0.82	0.96	0.86
	AES		2 ×$10^{-4}$	2 ×$10^{-3}$	5 ×$10^{-3}$	−8 ×$10^{-4}$	2 ×$10^{-3}$	6 ×$10^{-4}$	4 ×$10^{-4}$	4 ×$10^{-3}$	7 ×$10^{-4}$

**Table 6:** UACI test for different color images. Different values of *m* (m=7.5,8.8) and *T* (T=1,2,3) were tried respectively, where “lena” is Figure 22a, “boat” is Figure 22b, and “veg” is Figure 22c.

Object			UACI		
			R	G	B
lena	*m* = 7.5	*T* = 1	46.293217	40.515568	24.708355
		*T* = 2	43.678409	43.262431	37.007906
		*T* = 3	41.407206	42.583560	29.628579
	*m* = 8.8	*T* = 1	46.454520	41.186064	24.949227
		*T* = 2	44.341227	43.377668	33.920451
		*T* = 3	43.072341	40.273322	40.672362
	AES		50.133255	50.047464	49.936812
boat	*m* = 7.5	*T* = 1	35.143726	48.660561	52.421603
		*T* = 2	39.571601	55.066602	56.038358
		*T* = 3	47.716444	53.135069	53.060568
	*m* = 8.8	*T* = 1	35.245613	50.609906	55.037498
		*T* = 2	38.337034	56.456049	58.441035
		*T* = 3	42.331758	55.305545	56.647502
	AES		49.953939	50.052023	50.175874
veg	*m* = 7.5	*T* = 1	37.751291	54.587069	54.226224
		*T* = 2	45.768913	47.626056	62.656476
		*T* = 3	37.213291	48.628576	69.999379
	*m* = 8.8	*T* = 1	37.915899	54.778044	55.353727
		*T* = 2	62.202183	48.556424	40.446628
		*T* = 3	33.298991	51.570749	69.901463
	AES		49.956022	50.026667	49.943955

**Table 7:** Similarity comparison between the grayscale encrypted images I1 and I2 with the proposed approach. Six different encrypted images are taken, the similarity is calculated between each two, and no comparison is made between encrypted images of the same original image. The result column has three values, from top to bottom, corresponding to (47)–(49). In particular, “lena” is Figure 21a, “boat” is Figure 21b, and “clock” is Figure 21c.

	I2	Lena (*m* = 6, *T* = 1)	Boat (*m* = 7.5, *T* = 2)	Clock (*m* = 6, *T* = 2)
I1				
lena (*m* = 7.5, *T* = 1)			0.068396	0.0898650
			0.112057	0.119775
			0.999999	0.999999
boat (*m* = 7.5, *T* = 1)		0.042764		0.099446
		0.077088		0.143051
		0.999999 *		0.999999
clock (*m* = 6, *T* = 3)		0.106253	0.071040	
		0.107990	0.081516	
		0.999999	0.999999	

*: The value is 0.9999999977 when 10 decimal places are used.

**Table 8:** Similarity comparison between the color encrypted images I1 and I2 with the proposed approach. Six different encrypted images are taken, the similarity is calculated between each two, and no comparison is made between encrypted images of the same original image. The result column has three values, from top to bottom, corresponding to (47)–(49). In particular, “lena” is Figure 21a, “boat” is Figure 21b, and “veg” is Figure 21c.

	I2	Lena (*m* = 8.8, *T* = 2)	Boat (*m* = 7.5, *T* = 2)	Veg (*m* = 8.8, *T* = 1)
I1				
lena (*m* = 8.8, *T* = 3)			0.047194	0.070985
			0.103503	0.184824
			0.999999	0.999999
boat (*m* = 7.5, *T* = 1)		0.084080		0.008566
		0.185896		0.037862
		0.999999 *		0.999999
veg (*m* = 7.5, *T* = 1)		0.092913	0.106315	
		0.191360	0.210313	
		0.999999	0.999999	

*: The value is 0.9999999865 when 10 decimal places are used.

## Data Availability

The details of the data are described in Section 2.4 and Section 3.
